# Upconverting microgauges reveal intraluminal force dynamics *in vivo*

**Published:** 2025-10-09

**Authors:** Jason R. Casar, Claire A. McLellan, Cindy Shi, Ariel Stiber, Alice Lay, Chris Siefe, Abhinav Parakh, Malaya Gaerlan, Wendy Gu, Miriam B. Goodman, Jennifer A. Dionne

**Affiliations:** 1 Department of Applied Physics, Stanford University, Stanford CA, 94305, USA; 2 Materials Engineering Division, Lawrence Livermore National Laboratory, Livermore CA, 94550, USA; 3 Department of Biology, Stanford University, Stanford CA, 94305, USA; 4 Department of Mechanical Engineering, Stanford University, Stanford CA, 94305, USA; 5 Department of Molecular and Cellular Physiology, Stanford University, Stanford CA, 94305, USA; 6 Department of Radiology, Stanford University, Stanford CA, 94305, USA; 7 Department of Materials Science and Engineering, Stanford University, Stanford CA, 94305, USA; 8 Chan Zuckerberg Biohub, San Francisco, San Francisco, CA 94158

## Abstract

The forces generated by action potentials in muscle cells shuttle blood, food, and waste products throughout the body’s luminal structures. While non-invasive electrophysiological techniques exist,^1–3^ most mechanosensitive tools cannot access luminal structures non-invasively.^4–6^ Here, we create non-toxic, ingestible mechanosensors to enable the quantitative study of luminal forces and apply them to study feeding in living *Caenorhabditis elegans* roundworms. These optical “microgauges” comprise upconverting NaY_0.8_Yb_0.18_Er_0.02_F_4_@NaYF_4_ nanoparticles (UCNPs) embedded in polystyrene microspheres. Combining optical microscopy and atomic force microscopy to study microgauges *in vitro*, we show that force evokes a linear and hysteresis-free change in the ratio of emitted red to green light. With fluorescence imaging and non-invasive electrophysiology, we show that adult *C. elegans* generate bite forces during feeding on the order of 10 μN and that the temporal pattern of force generation is aligned with muscle activity in the feeding organ. Moreover, the bite force we measure corresponds to Hertzian contact stresses within the pressure range used to lyse the worm’s bacterial food.^7,8^ Microgauges have the potential to enable quantitative studies that investigate how neuromuscular stresses are affected by aging, genetic mutations, and drug treatments in this and other luminal organs.

Hollow neuromuscular organs, such as those of the cardiovascular and gastrointestinal systems, rely on electrical induction of muscle activity to generate force and propel material. Dysfunction in the magnitude, frequency, or coordination of force generation is a hallmark of motility diseases. For example, contractile pathologies can affect bolus transport in the esophagus,^9^ bladder function in patients with nervous system damage,^10^ and heart rhythms in patients with inherited arrhythmia syndromes.^11^ Unfortunately, a quantitative understanding of the local pressure gradients generated by electrical signaling remains incomplete due to a lack of miniaturized mechanosensing devices to access neuromuscular cavities non-invasively. The study of force in biology has been advanced by techniques such as optical tweezers, atomic force microscopy (AFM), and traction force microscopy (TFM), but these instruments cannot access *in vivo* tissues.^4,12^ Catheter-based techniques such as manometry^13,14^ are limited to larger organs, and while micromachined mechanosensors show promise,^6^ their use requires surgical implantation. Fluorescent nanomaterials, including Förster resonance energy transfer-based strain sensors,^15^ can overcome some constraints imposed by luminal organs due to their small size and optical readout modality. Indeed, such sensors have led to remarkable insights into mechanosensation from the cellular^16,17^ to the organism level.^18^ Their dynamic range (~1–10 pN) generally limits their utility to stresses induced on or by proteins.^5^ Moreover, the propensity of fluorophores to rapidly photobleach makes tracking force actuation over minutes a difficult task, and their excitation by visible light creates strong autofluorescence, decreasing their signal-to-noise ratio. To date, the field of mechanobiology lacks techniques that are well suited for measuring the relatively large compressive forces collectively actuated by many muscle cells inside the internal, narrow, and tortuous structures of the body’s tubular cavities.

Mechanosensitive upconverting nanoparticles (UCNPs) are a class of biocompatible^19–21^ materials that have been explored for next-generation optogenetics^22^ and phototherapy^23^ applications. Their popularity in biological applications stems from their ability to generate visible emission from low-energy infrared excitation, which induces negligible autofluorescence background and penetrates deeply into tissues.^24^ The distinct anti-Stokes shift is possible via the sequential non-radiative energy transfer (ET) between long-lived 4f states of trivalent lanthanide dopant pairs.^25^ Our group has previously demonstrated the ratiometric sensitivity of red (^4^F_9/2_ → ^4^I_15/2_) and green (^2^H_11/2_ +^4^S_3/2_ → ^4^I_15/2_) emission lines (Fig. 1a) within core@shell cubic-phase (*α*) NaY_0.8_Yb_0.18_Er_0.02_F_4_@NaYF_4_ UCNPs to (quasi)hydrostatic pressure.^26–28^ This material exhibits an optimal balance between high sensitivity and brightness, undergoing a visible color change that can be registered on a standard RGB camera.^27^

We develop *α*-NaYF_4_ UCNP-embedded polystyrene microbeads (referred to as “microgauges” below) that are sensitive to micronewtons of uniaxial compressive force, and we calibrate their mechano-optical response using dual confocal optical and atomic force microscopy (confocal AFM). To demonstrate their utility, we use the *C. elegans* nematode pharynx, a luminal neuromuscular organ responsible for the mechanical digestion of bacterial food. The action and mechanisms of pharyngeal pumping, the animal’s method of feeding, bear striking similarities to those of the human heart, including homologous ion channels that regulate action potentials.^29–31^ Combined with the ease of *C. elegans* cultivation and fluorescence imaging, these factors make pharyngeal pumping an attractive model behavior for sensor demonstration.^32^ The polystyrene vessel mimics the size of the worm’s bacterial food source, allowing it to bypass a size-selective filtering mechanism after ingestion, and provides a controlled ET environment during luminal transit. Ingested microgauges exhibit no toxicity and respond to increasing compressive forces with an increase in their red-to-green emission ratio, which we record in concert with the action potentials that regulate feeding. We observe a temporal correlation between the electrical induction of contraction and the ratiometric emission profile in the pharyngeal lumen of live *C. elegans* roundworms and find that this tissue generates an average maximal force increase on the order of 10μN. This system utilizes correlated electrophysiology and mechanical imaging in live organisms to directly measure bite force in *C. elegans*. Microgauges offer a framework for linking genetic disorder to defects in force generation in neuromuscular organs. However, their future application is not limited to *C. elegans*, as microgauges can be customized to other nematodes and other animals.

Core@shell *α*-NaY_0.8_Yb_0.18_Er_0.02_F_4_@NaYF_4_ UCNPs are synthesized via thermal decomposition and hot injection.^28^ Trivalent ytterbium and erbium dopants in the nanoparticle cores cooperatively convert incident 980 nm near-infrared excitation into visible emission with two prominent lines in the red (660 nm) and green (520 + 545 nm) spectral regions (Fig. 1a,d). The inert NaYF_4_ shell decreases the ET rate from dopants in the core to vibrational modes on the solvent, enhancing the UCL intensity.^33^ Brightness is further enhanced once the UCNPs are packaged in hydrophobic polystyrene, especially when delivered to the water-filled pharynx. Aqueous environments pose a significant challenge to UCNP sensor performance for several reasons: water can rapidly disintegrate the lattice,^34^ its high-energy -OH vibrational modes efficiently quench UCL,^35^ and aqueous solutes can affect the emission color, brightness, and lifetime in ways that might compete with mechanical stimuli.^36^ We avoid this signal loss and stimulus competition by packaging UCNPs in a controlled chemical environment via a modified emulsion polymerization method,^37^ as depicted in Fig. 1b. This procedure results in UCNPs that are randomly and densely distributed throughout the interior of the polymer host, as revealed by transmission electron microscopy (TEM) images of whole microgauges (Fig. 1b) and scanning electron microscopy (SEM) images of ion-milled microgauge cross sections (Fig. 1c, SI Video 1). The peak energies of the microgauge UCL spectra are identical to those of the constituent UCNPs, but there is a small decrease in the relative proportion of green photon emission (Fig. 1d). This is likely due to the presence of polystyrene, the aromatic -CH stretching modes of which have better resonance with the green quenching ^4^S_3/2_ → ^4^F_9/2_ transition than the red quenching ^4^F_9/2_ → ^4^I_9/2_ transition (ED Fig. 4). Overall, this encapsulation procedure affords UCNPs a brightness that is consistent and sufficient to image with high temporal resolution (20 ms), as detailed in later sections.

In addition to maximizing brightness in the aqueous environment of the lumen, this encapsulation allows UCNPs to bypass the size-selective filtering mechanism of the pharynx, which will eject material smaller than ~200 nm.^38^ Like the human heart, the pharynx is a semi-autonomous neuromuscular organ, which rhythmically contracts and relaxes in response to action potentials.^30^ In the corpus and anterior isthmus, the outward radial contraction of epithelial muscles opens the pharyngeal lumen, drawing particle-laden fluid in through the buccal cavity (Fig. 1f). The subsequent relaxation of these muscles seals the lumen, retaining large particles at trap sites in the anterior corpus (procorpus) and anterior isthmus while ejecting fluid and smaller particles. The procorpus cross sections in Fig. 1f depict how this is achieved. Small material is diverted radially outward through narrow constrictions between the central lumen and apical channels, where it can bypass the trap sites. Microgauges are too large to be expelled through these constrictions but not so large that they cannot enter through the buccal cavity (~3 μm).^38^ Seeking to mimic the primary target of this filtering mechanism, *E. coli* (Fig. 1e), we engineer an average microgauge size of 935 nm (Extended Data Fig. 1). As expected, *C. elegans* allowed to freely ingest Poly(maleic anhydride-alt-1-octadecene) (PMAO)-wrapped UCNPs displayed markedly lower and less frequent UCL intensities than those incubated with microgauge-embedded UCNPs (Extended Data Fig. 2). The pharynx, with its aqueous environment and filtering mechanics, imposes crucial design constraints on UCNP-based mechanosensors, which are efficiently satisfied with the microgauge construct.

## Biocompatibility

**Figure 2:**
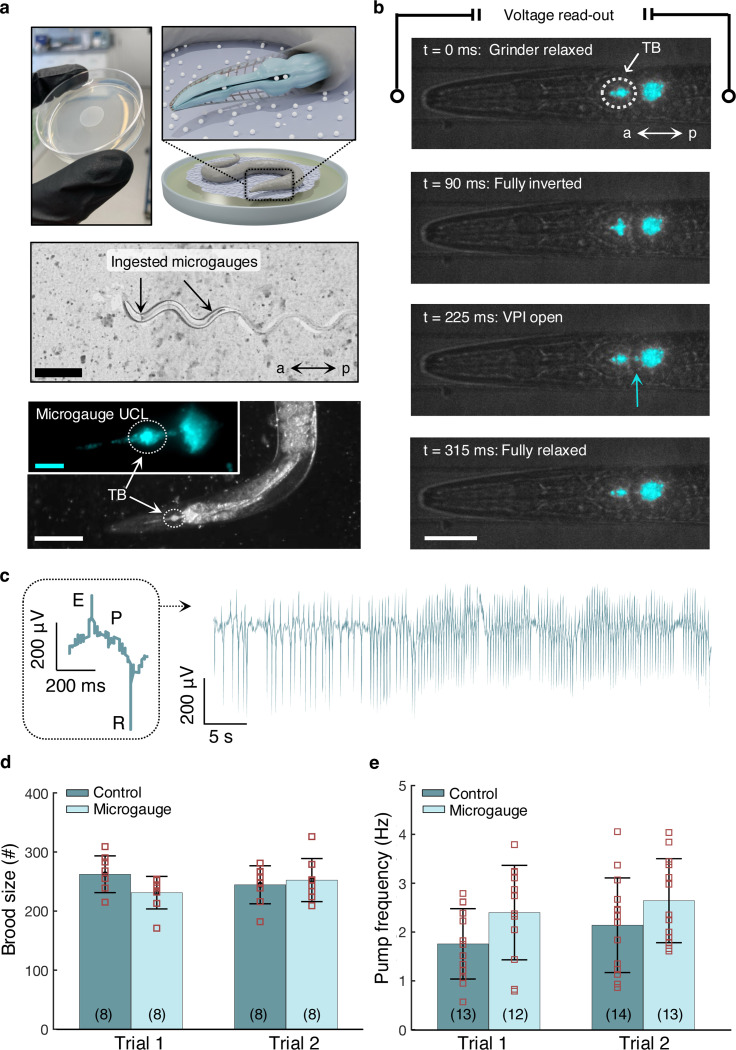
*Caenorhabditis elegans* ingest biocompatible microgauges **a)** Top, an agar plate and microgauge lawn, a schematic representation of a worm consuming microgauges from this lawn and a pharynx highlighting the lumen containing ingested microgauges. Middle, an image of a worm on this lawn. Microgauges (dark contrast) are visible in the upper and lower intestines. Bottom, false-colored UCL (cyan) and corresponding brightfield images from ingested microgauges in the pharynx and upper intestines. Scale bars, 250 μm (top), 100 μm (bottom) and 20 μm (inset). **b**) Brightfield and false-colored UCL (cyan) stills of particle transport during serotonin-stimulated pumping for a worm inside the immobilization channel of the EPG chip. Particle transport through the vpi is visible in the third frame and denoted with a blue arrow (source: Supplementary Video 2). Scale bar, 30 μm. **c**) A full 60s EPG time series and waveform (inset) highlighting depolarization (E, contraction onset), plateau (P, contraction maintenance), and repolarization (R, relaxation onset) signal from pharyngeal muscles. **d**) Average brood size (mean ± s.d.) of animals fed *E. coli* with microgauges (light blue; trial 1: 231 ± 28; trial 2: 252 ± 36) or without microgauges (dark blue, trial 1: 262 ± 31; trial 2: 245 ± 32). There was no statistically significant effect of microgauges (trial 1: *p =* 0.09 and trial 2: *p* = 0.70, Student’s t-test). Orange circles are results from individual animals, and error bars are standard deviations. **f**) Average pharyngeal pumping rate (mean ± s.d.) from animals fed *E. coli* with microgauges (light blue; trial 1: 2.40 ± 0.97 Hz; trial 2: 2.64 ± 0.86 Hz) or without microgauges (dark blue; trial 1: 1.76 ± 0.72 Hz; trial 2: 2.14 ± 0.97 Hz). There was no significant effect of microgauges (trial 1: *p =* 0.09 and trial 2: *p = 0.17*, Student’s t-test). The red squares are the average pharyngeal pumping rates for individual worms over a 60s window, and the error bars are their respective standard deviations. VPI = pharyngeal intestinal valve, a = anterior, p = posterior, TB = terminal bulb.

Having constrained the sensor size to mimic the worm’s bacterial food source, we can co-opt its natural feeding behavior to passively localize our sensors in the terminal bulb. Figure 2a summarizes the feeding procedure, in which adult animals consume both *E. coli* and microgauges from a small lawn on agar. Microgauge sensors that pass through the pharynx into the intestine are expelled via defecation. There are no obvious differences in animal movement, feeding, or defecation when microgauges are present (Supplementary Videos 3–5). Although their accumulation varies between individuals, microgauges typically accumulate in the anterior isthmus and terminal bulb (Fig. 2a), which is consistent with the current understanding of particle transport in the pharynx.^39^

The grinder resides in the terminal bulb and consists of three radially oriented cells with ridged cuticles. It inverts to mechanically degrade the cell walls of trapped bacterial food.^40^ This inversion is nearly synchronous with the onset of contraction in the corpus. Normal transport dynamics during a single representative pump are illustrated in dual UCL (cyan) and bright field video frames (Fig. 2b, Supplementary Video 2). Between frames one and two, the grinder goes from fully relaxed to fully inverted, forcing the microgauges within the terminal bulb posteriorly. Microgauge transport through the partially open pharyngeal intestinal valve (VPI) into the intestines is visible at the blue arrow in the third frame. The pharyngeal muscles then relax, returning the grinder to a resting position. The imaging rate (66 frames per second) is more than 10x the maximum frequency of pharyngeal pumping (~5 Hz). We note that imaging at this rate requires a high irradiance (13 kW/cm^2^), which induces minor heat stress in the pharynx (Supplementary Fig. 1).

In the first of two biocompatibility assays, we study the chronic toxicity and reproductive effects of microgauges by measuring the total progeny generated during the four-day window from egg-laying onset until cessation (Fig. 2d). In both trials, the fecundity of worms fed a standard diet of *E. coli* are indistinguishable from that of worms fed a diet of *E. coli* mixed with microgauges (Supplementary Fig. 2). Moreover, both the daily number of progeny and the total brood size between the two conditions are similar to those of wild-type worms fed a normal bacterial diet.^41^ Similarly, a previous study has found no effect of unencapsulated, ligand-stripped *α*-NaYF_4_ on worm fecundity,^21^ suggesting that UCNPs are non-toxic whether delivered alone or encapsulated in polystyrene.

In the second assay, we measure the effects of microgauge ingestion on pumping frequency, which is estimated non-invasively from an electropharyngeogram (EPG). The EPG reflects the pattern of muscle contractions associated with pumping and can be used to determine their frequency. To measure EPGs, young adult worms were loaded into a specialized microfluidics device and treated with exogenous serotonin to stimulate pumping according to established procedures.^11,42^ The inset in Fig. 2c shows the waveform corresponding to a single pump, consisting of an excitation phase (E), followed by a repolarization phase (R). The former triggers the contraction of the anterior pharyngeal muscles - and the terminal bulb muscles with a slight delay - and the latter triggers their subsequent relaxation. Across two trials, the average pharyngeal pumping rate of animals fed a normal bacterial diet is indistinguishable from that of animals fed bacteria plus microgauges (Fig. 2e). Thus, microgauge ingestion and accumulation do not alter pharyngeal function.

## Mechano-optical calibration

**Figure 3:**
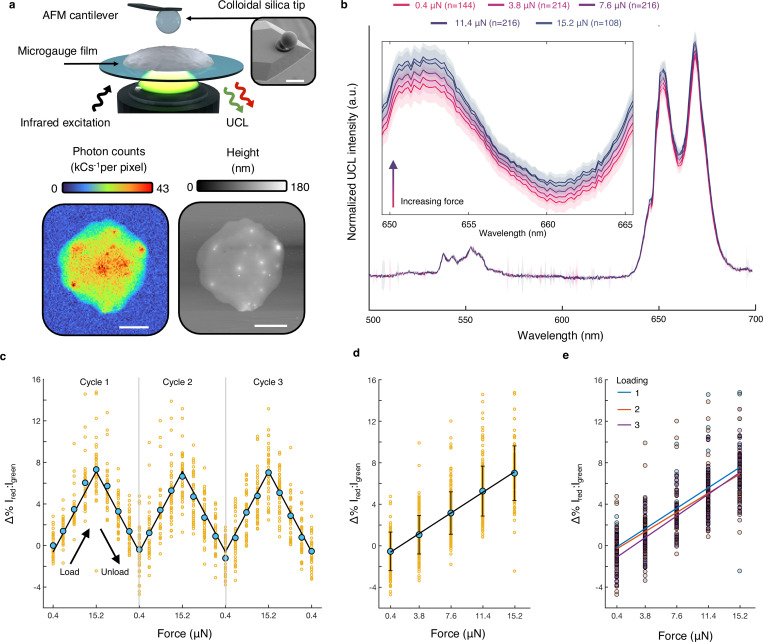
Optical mechanosensitivity is calibrated using confocal AFM **a**) Top, a schematic of the calibration setup. An objective below the coverslip-mounted microgauge film simultaneously excites and collects UCL for imaging or spectroscopy. Inset, an SEM image of the indentation tip. Bottom, two coincident images of a sample film: UCL photon counts arriving at a single photon counting module (left), and film height measured in AC mode with a 30 nm radius rounded silicon tip (right). **b**) Averages of 898 green-normalized UCL emission spectra grouped by indentation force. The dark lines represent spectra averages, and lighter regions represent the standard deviation of the wavelength. Inset, an enlarged red peak. **c**) The percent change in the ratio of integrated emission intensities as the film is indented between 0.4 and 15.2 μN in three consecutive cycles. Shown are individual ratio changes from the 12 replicate locations (orange), the averages for each force at a particular order in the loading-unloading cycle (cyan) and the best fit line (black). **d**) The data in **c** grouped by force. **e**) The data in **d**, colored by cycle number, with best-fit lines for each cycle. No statistically significant differences in the slopes of best fit lines are observed at a 95% confidence level. (*p*_1,2_ = 0.49; *p*_1,3_ = 0.45, n_1_ = n_3_ = 180; n_2_ = 179).

Next, we sought to determine how local, anisotropic compressive loads affect UCL emission color. By integrating laser-scanning confocal microscopy and AFM, performed simultaneous imaging and spectroscopy (Fig. 3a, Supplementary Figs. 4–6). These measurements used anisotropic forces on the order of micronewtons applied across spatial scales that match those of the feeding organ, a choice intended to replicate the likely biological mechanics. The goal of mechanical digestion of bacteria in the terminal bulb is to maximize nutrient retrieval and prevent bacterial colonization of the gut. Although hydrostatic stress may increase slightly as isthmus peristalsis^39^ moves material into the sealed compartment of the terminal bulb, the primary stresses used to lyse the bacterial cell walls are anisotropic (compression and shear) and imparted by the grinder. Underlying this assertion is the fact that the guts of worms with defective grinders exhibit much higher live bacterial loads than the wild type.^43–45^

The range of forces used for calibration (0.4 to 15.2 μN) is meant to approximate inactivation stresses *in vivo*. Approximately 1 in 1,000 *E. coli* per hour survive to colonize the intestines,^46^ a rate which reflects both the efficacy of the grinder and the ability of unlysed bacteria to adhere to the intestinal wall. Depending on operating conditions, high pressure homogenizers achieve similar lethality rates for *E. coli* between 50 and 200 MPa.^7,8^ Informed by these bounds, we employ a Hertzian contact model^47^ based on geometrical and material assumptions about *E. coli*,^48^ and the cuticular ridges of the grinder^40^ to calculate an equivalent uniaxial compressive force in the range of micronewtons to 10s of micronewtons. This conversion also requires knowledge of the compressive modulus of the microgauges (740 MPa), which we derive by fitting 50 single microgauge force indentation curves to a Johnson-Kendall-Roberts (JKR) model (Supplementary Fig. 7).^49^ We constrain the upper bound of calibration stress (42 MPa) to less than half of the yield strength of microscale polystyrene (~100 MPa).^50^ As expected, we observe no plastic deformation in samples indented under these conditions (Supplementary Fig. 8). Surprisingly, we do not observe permanent divoting in SEM images until indentations exceeding 400 MPa equivalent stress (Supplementary Fig. 9), which could be due to the presence of significantly stiffer ceramic nanoparticles in this composite material. Using a confocal AFM to calibrate local UCL changes against micronewton-scale anisotropic compressive forces, we more faithfully replicate the mechanical conditions that likely exist *in vivo*.

Compressive loads between 0.4 and 15.2 μN result in a linear increase in the ratio of integrated red (634–689 nm) and green (513–566 nm) emission (Fig. 3b). To ensure sample stability during loading-unloading cycles, we sampled thin films formed from microgauge monolayers (Extended Data Fig. 3), and to ensure that the confocal excitation spot collects UCL exclusively from the contact area, we indent with a large 10.2 μm diameter spherical silica tip. Figure 3d shows the change in emission ratio as a function of applied force, which is well fitted by a linear model with slope 0.52% I_Red_:I_Green_ per micronewtons (s.e.m. = 0.02%). Enhancement of select phonon-mediated (Extended Data Fig. 4) and cross relaxation-mediated (Supplementary Fig. 12) quenching pathways may be responsible for the stress-induced shift toward redder emission. The observed AFM trends are consistent with the linear colorimetric trends reported for various UCNPs under hydrostatic pressure in diamond anvil cell (DAC) studies.^21,26–28^ DAC experiments on our microgauges (Extended Data Fig. 5) yield a similar, though dampened, pressure response (5.6% Δ%I_Red_:I_Green_/GPa, vs. 9.2% measured previously for unembedded UCNPs).^28^ DAC-measured hydrostatic pressure sensitivity can be converted to an approximate force sensitivity (5.4% Δ%I_Red_:I_Green_/μN) using several simplifying assumptions (Methods). Deviations from these assumptions, as well as the distinction between isotropic stresses obtained in the DAC and anisotropic stress delivered via AFM, may account for the tenfold discrepancy observed.

Confocal AFM measured force responses are mechanically and optically robust. At the average irradiance used in our experiments (400 kW/cm^2^), variations in power density due to laser output produce a change in I_Red_:I_Green_ of 0.018% (Supplementary Fig. 11), which is below the error in force sensitivity. Responsiveness is independent of UCNP loading (Supplementary Fig. 10). Moreover, the sensitivity is consistent when cycled three times within the elastic regime of polystyrene (Fig. 3c). To verify this, we compare the second and third loadings to the initial loading using two-tailed t-tests (Fig. 3e). No statistically significant differences in the slopes of best-fit lines are observed at a 95% confidence level. Given that replicate measurements are taken sequentially over ~2 hours of continuous laser exposure, the lack of hysteresis also suggests the response is not induced by temperature. Considering the rhythmic pattern of compressions exerted by pharyngeal muscles - and semi-autonomous neuromuscular organs more generally - these data suggest that the mechanical and optical properties of the microgauges will be independent of their loading history.

## Electrophysiology and mechanical imaging

**Figure 4:**
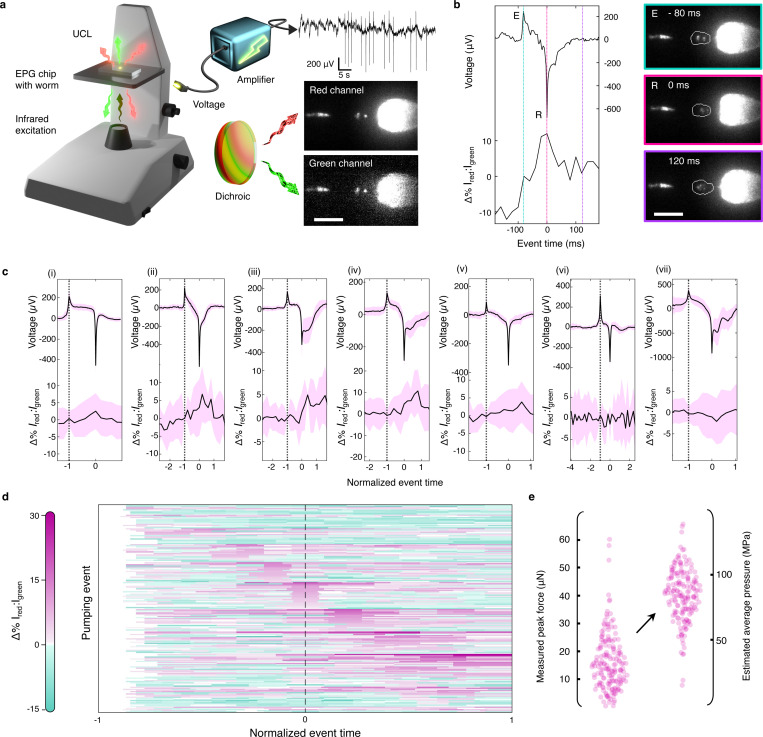
Microgauges enable simultaneous electrophysiology and optical mechanical imaging of feeding forces in live worms **a**) A schematic illustrating the correlated electrical and opto-mechanical recording setup, in which UCL luminescence is collected from microgauges within immobilized worms, and then separated into red and green channels (3,000 frames, 50 fps). Example UCL frames and EPG traces correspond to the data set in **c.ii**. Scale bar, 30 μm. **b**) Electrical and optical data for one representative pump, along with the red frames corresponding to the highlighted timepoints. The white outlines surrounding the terminal bulb represent the boundaries of the pixel segments used to extract Δ%I_red_:I_green_. Scale bar, 30 μm. **c)** Event-triggered averages of EPG waveform and Δ% I_red_:I_green_ for all pumps in the 60s recording window, for each of seven worms. Plots i-vii represent each individual animal (n_i_ = 70, n_ii_ = 13, n_iii_ = 12, n_iv_ = 18, n_v_ = 29, n_vi_ = 18, n_vii_ = 25). Magenta indicates the standard deviation of each normalized time point. **d**) A raster plot of each time-normalized pumping event sorted from top to bottom according to the relative timing of the maximal Δ% I_red_:I_green_ (n = 185). Note that the ratio change at each time point is normalized to the value recorded at E, so all pixels corresponding to E have a value of +0% I_red_:I_green_ (white). **e**) The maximum force change exhibited over each of the 169 event windows with a detectable force increase (mean ± s.d. = 15.7 ± 10.1 μN), as well as their corresponding average contact pressures (84 ± 20 MPa) converted using a Hertz model assuming a 1 GPa grinder stiffness and an effective contact radius equal to the average microgauge radius, as described in Supplementary Information Section 4.

To demonstrate that these microgauges monitor intraluminal force dynamics *in vivo* and to obtain a quantitative estimate of *C. elegans* bite forces, 60s epochs of correlated two-channel UCL imaging and electrophysiological data were collected from wild-type worms in tandem (Fig. 4a). From these data streams, we obtained traces of the ratio of red and green emission intensities from the terminal bulb of the pharynx (force traces) and of the electrical activity of muscles in the pharynx (EPG), as described (Supplementary Figs. 13–16, Section 7). Individual pharyngeal pumping events are detected in the EPG signal and aligned to the corresponding portion of the force trace (Fig. 4b, Supplementary Fig. 13). As expected from prior studies of pharynx-induced particle movements,^38,39^ microgauges are pushed posteriorly during the E-to-R interval, reverse direction after the R phase, and cease moving ~100 ms later (Fig. 4b, Supplementary Video 6).

Each 60s epoch contained dozens of similar pumping cycles, which we leverage to generate event-triggered averages of both EPG and force signals. Because the time separating the E and R phases is variable, it is necessary to align events to the R phase (*t* = 0) and to normalize time to the duration between the E and R phases (see Supplementary Information Section 7). Figure 4c shows event-triggered EPG averages and the corresponding normalized force traces for seven worms. In five worms (i-v), average Δ%I_Red_:I_Green_ values increase within the terminal bulb following contraction. One worm (Fig. 4c.vi) has no detectable change in force and a very short average pump duration (52 vs. the 111 ms average across the other six animals), and another exhibits a modest decrease (Fig. 4c.vii). We note that force traces are derived from the emission of multiple microgauge sensors as they pass through the terminal bulb as the worm feeds, a factor that may contribute to the variation evident in the event-triggered average force traces (Fig. 4c) and across the 185 individual events studied here (Fig. 4d). Very few events (16) lacked a detectable force increase and most (169 of 185) contained a force increase within 100 ms of the R phase of the EPG. In approximately one-third of these cases (60 of 169), the event maximum occurred within 20 ms of the relaxation of the corpus or terminal bulb. This temporal pattern of changes in force is consistent with that of the muscle contractions that govern pharyngeal pumping. Collectively, these findings indicate that microgauges detect the influence of muscle activity on intraluminal stress.

The peak change in the microgauge emission ratio, Δ%I_Red_:I_Green_, is 8.2 ± 5.2%, on average (Fig. 4e). These values are converted into a relative force increase (Fig. 4e) based upon our confocal AFM calibration (Fig. 3d), yielding an average maximum force increase of 15.7 ± 10.1 μN. To put this measurement of nematode bite force in context, it is useful to consider that the central function of the terminal bulb and the grinder is to enable nutrient retrieval and to minimize bacterial colonization of the worm’s gut. The mean value of average Hertzian contact stress estimated from each peak force (84 ± 20 MPa, as seen in Fig. 4e and described in Supplementary Information Section 4) is consistent with that used in laboratory pressure vessels^8^ to achieve a 1,000-fold inactivation of bacterial growth, which is similar to what is observed in living worms.^46^ Thus, the stress generated during each pump seems to be sufficient to subserve the prime function of the nematode pharynx.

In summary, we have synthesized, calibrated, and deployed *in vivo* an optically readable sensor of mechanical compression in the lumen of hollow neuromuscular organs. We have demonstrated its viability in quantifying forces associated with pumping in the *C. elegans* pharynx, which is a promising model system for studying arrhythmia and hypercontractility. We envision that this platform could enable the direct comparison of luminal force generation in worms with dysregulation of the duration or frequency of contraction-inducing action potentials in calcium channel mutants^31,39^ and how they respond to drug treatment.^29,30^ This platform may also find utility as a quantitative, functional replacement for lower-throughput structural assays of muscle mass loss^51^ to expedite the study of the effects of caloric restriction, aging, and drug intervention on sarcopenia and healthspan.^52^ This proof-of-concept demonstration reveals several opportunities to increase microgauge signals and reduce noise. Utilizing a brighter material with a lower ambient I_Red_:I_Green_ ratio - such as the “fully-doped” SrYb_0.72_Er_0.28_F_5_@SrLuF_5_ - will improve the signal-to-noise ratio of the green emission channel^27^ and enable the use of lower excitation power densities to avoid unwanted heating over extended imaging periods. Improved polymerization methods that narrow the distribution of sensor size and UCNP loading density, along with a continuous, in-chip feeding procedure, will help eliminate inconsistencies in absorption and emission cross sections as the worm cycles particles through its terminal bulb. With these improvements, this assay has the potential to directly and non-invasively distinguish intraluminal contractile capability between cohorts of animals and provide insights into the interplay between electrical control and mechanical efficacy.

## Methods

### Upconverting nanoparticle synthesis

The NaY_0.8_Yb_0.18_Er_0.02_F_4_@NaYF_4_ UCNPs used to make microgauges were prepared, washed, and stored according to previously reported methods.^28^

### Emulsion polymerization

This procedure was modified from an existing method.^37^ Dried UCNPs (15 mg) were resuspended in styrene (1 mL, stabilized, 99%, Acros) and then emulsified in a 5.6 mL aqueous solution of sodium dodecyl sulfate (SDS, 1 mM) and potassium persulfate (KPS, 6 mM) via brief sonication (~2 min). Polymerization of UCNP/styrene micelles into microgauges was achieved after 20 hours of continuous stirring (330 RPM) and heating (66°C) in a vented 20 mL scintillation vial (22G needle). The product was washed in a centrifuge (1000 RCF, 10 minutes, 2x) and allowed to sediment without agitation over a 24-hour period in order to separate the denser microgauges from most of the unembedded or sparsely embedded polymer.

### UCNP surface modification for aqueous dispersal

Aqueous dispersions of single nanoparticles were achieved by wrapping the hydrophobic oleic acid-capped UCNPs with Poly(maleic anhydride-alt-1-octadecene) (PMAO) (Extended Data Fig. 2d). This scheme was modified from an existing method^53^ to include 4-dimethylaminopyridine (DMAP, Thermo Fisher Scientific) as an anhydride esterification catalyst, rendering the PMAO more amphiphilic. Dried UCNPs (5 mg) and PMAO (165 mg, Sigma Aldrich Cat. #419117) were first suspended in dichloromethane (DCM, 530 μL ) and then combined with MilliQ water (10 mL, Merck-Millipore) and DMAP stock solution (185 μL, 400 mM in DCM). After 30 minutes of sonication, the milky white suspension was transferred to a 50°C oil bath to evaporate the DCM. Once clear and viscous, PMAO-wrapped UCNPs were washed of excess polymer and catalyst in an Amicon-Ultra15 centrifugal filter (100 kDa cutoff) spun at 5,000 RPM (5 min, 3x). The retentate was suspended and stored in 1 mL MilliQ water.

### Microgauge film preparation

Approximately 10 μg of microgauges were dropcast from suspension onto a plasma hydrophilized silica 0.17 mm thick coverslip (for mechano-optical calibration) or silicon wafer chip (for SEM imaging) and spun at 500 RPM for 30–90s until dry. The resulting microgauge monolayers were placed in a preheated 290°C oven for 30 minutes to allow the polystyrene matrix to melt and then rapidly cooled through the glass transition temperature to room temperature. Most film patches exhibited a gently domed structure that ranged from a minimum of 50 nm thickness at the edges to a maximum of 600 nm thickness at the center.

### Electron microscopy

A transmission electron microscope (Tecnai G2 F20 X-TWIN, Thermo Fisher Scientific) operating at 200 kV was used to image core and core@shell nanoparticles. Size distributions were measured manually with the open-source image analysis software FIJI.^54^ Similarly, size distributions for dropcast microgauges were measured using SEM images taken with a scanning electron microscope (Apreo S LoVac, ThermoFisher Inc.) operating at 5kV and 50 pA. This instrument was also used to image uncoated microgauge monolayers before and after melting and to visualize film surfaces before and after indentation. A focused ion beam scanning electron microscope (FEI Helios Nanolab 600i DualBeam FIB/SEM, Thermo Fisher Scientific) equipped with a Tomahawk Ga^+^ ion beam was used to assess the 3D distribution of UCNPs in individual microgauge cross sections. In the first step, a conductive platinum layer (500 nm, 51.76 pA) was deposited, and the microgauge was milled enough to expose the interior for focusing. After refocusing, the remainder of the microgauge was alternatively milled (30 kV, 40 pA, 2 μm depth) and imaged (2 kV, 170 pA) with real-time end-point monitoring.

### Confocal atomic force microscopy

An atomic force microscope head (MFP-3D, Oxford Instruments) fitted with a 10.2 μm diameter spherical silica tip (sQube, CP-NCH-SiO-D5) was mounted on the stage of a microscope (Zeiss Axio Observer) and sealed in an acoustic isolation chamber (Ametek TMC Vibration Control). The spring constant of the cantilever (24.29 nN/nm) was calibrated using the thermal method within the manufacturer’s software, and the inverse optical lever sensitivity (155.96 nm/V) was calibrated against a bare silicon coverslip. These values were used to calculate the appropriate voltage trigger for each force. The respective voltage was held for three consecutive 90s spectral acquisitions on a spectrometer (Isoplane SCT-320; 500 nm 300 gr/mm grating) fitted with a digital camera (Blaze 400-LD, Teledyne Instruments). Any spectra affected by cosmic rays within the red or green integration regions were reacquired before advancing to the next voltage trigger. Excitation from a 100 mW 980 nm Obis laser (Coherent Corp.) was focused through a Zeiss 40× 0.95 NA Plan Apochromat objective (400 kW/cm^2^). Within the collection path of the confocal optical train (Supplementary Fig. 4), two co-aligned single-mode fibers (SM980, 11 mm paf2p-11a collimators, Thorlabs) downstream of a 50:50 beamsplitter cube (CCM1-BS013/M, Thorlabs) acted as pinholes (Supplementary Fig. 5). The “transmission” fiber was used for spectroscopy, and the “reflection” fiber was coupled to a single photon counting module (Excelitas Technologies) for simultaneous imaging. Confocal images were generated by rastering the excitation beam across the sample with a fast-scanning mirror (Newport). Mirror control and fluorescence collection were timed under the control of the imagScan software package (provided by the laboratory of A. Jayich). Contrast differences between the indented (0.4 μN) and unindented microgauge samples were used to place the excitation spot at the center of the tip-sample contact region (Supplementary Fig. 6). To confirm that the excitation power density remained constant throughout the experiment, the axial stability of this coincidence was monitored in real time during the spectral acquisition by monitoring the counts received on the single photon counting module through the second (“reflection”) channel, and the lateral stability was confirmed at the end of each cycle by rescanning the confocal image. The percentage change in I_Red_:I_Green_ extracted from these spectra was referenced to the value at the initial 0.4 μN indentation rather than at the unstrained state to account for tip-and cantilever-induced field enhancements.^55^ Twelve non-overlapping locations were analysed with this three-cycle ramp.

### Force indentation curves

A parabolic silicon tip (SD-R30-FM-10, *r*=30 nm, 75 Hz, 2.8 nN/nm) operating in AC mode was used to locate 50 individual microgauges on a glass coverslip. Once located, each microgauge was indented in contact mode up to a 100 nN trigger force at a speed of 1 μm/s, and the force indentation curve was recorded. Indentation is calculated from the difference between the piezo displacement and the cantilever deflection, and force is calculated from the cantilever deflection according to Hooke’s law. Baseline corrections of the force-indentation curves were performed automatically within the manufacturer’s software. Gradient descent was used to estimate a compressive modulus by fitting the approach curves to the Johnson-Kendall-Roberts model (Supplementary Fig. 7).^49^

### DAC preparation for Raman and UCL measurement

To prepare the diamond anvil cell, a flake of dried microgauges and silicone oil as a high-pressure medium were loaded into a 300 μm diameter hole drilled using an electrical discharge machining dtool into a T301 stainless steel gasket between two 500 μm diameter diamond culets. Ruby powder was co-loaded as an internal pressure calibrant. After tightening or loosening the set screws, pressure was equilibrated for 30 minutes before acquiring spectra. Both microgauge UCL and ruby photoluminescence were captured on an inverted microscope (Zeiss Axio Observer) fitted with a 20× 0.42 NA 200 mm objective (Mitutoyo), a spectrometer (Princeton Instruments Acton 2500) and a digital camera (Princeton Instruments ProEM eXcelon). Microgauge UCL was excited with a 980 nm diode laser (OptoEngine MDL-III-980, 200 W/cm^2^), and spectra were acquired with a 500 nm 150 gr/mm grating, a 250 μm wide slit, and a 50s integration time. Ruby photoluminescence was excited with a tunable argon-ion laser at 488 nm (Innova 70c, 900 mW/cm^2^, Coherent Corp.), and spectra were acquired for 5s with the same spectrometer (Acton 2500; 500 nm 1,800 gr/mm grating, slit width 50 μm). Pressure (in GPa), was calibrated from the fluorescence line shift of the ruby R2 peak according to the relationship

Eq.1 $$\sigma =\frac{\mathrm{1904}}{7.\mathrm{715}}({\frac{\lambda }{\lambda_{0}}}^{7.\mathrm{715}}-1)$$

against an ambient pressure reference wavelength of $\lambda_{0}=694.38\text{nm}$.^56^ The cell was cycled three times consecutively between target minimum and maximum pressures of 0.0001 GPa (ambient) and 6 GPa, respectively. Each cycle consisted of five pressures during loading and one pressure during unloading. Red and green regions of the background-corrected spectra were integrated and the change in their ratio was referenced to the ambient pressure value.

DAC-measured pressure sensitivity was converted into an approximate force sensitivity as outlined previously.^26^ Assuming the UCNPs are perfectly spherical with uniform radii equal to the average radius, $r_{0}=9.1\;nm$, experience perfectly isotropic stress, σ, and behave with linear, isotropic elasticity, UCNPs will experience a total force according to

Eq.2 $$F(\sigma )=4\pi r_{0}^{2}\left(\frac{1}{E^{2}}\sigma^{3}-\frac{2}{E}\sigma^{2}+\sigma \right),$$

where the compressive modulus, E = 272 GPa, was measured previously.^26^ We stress that the three assumptions outlined above neglect random error due to UCNP size (coefficient of variation 9%), systematic error in surface area estimates due to non-spherical morphologies, the hydrostatic pressure limit for the silicone oil pressure medium (~3 GPa),^57^ and the presence of polystyrene as an effective pressure medium. The presence of polystyrene is particularly consequential, because the solid polystyrene will not propagate hydrostatic stress to its interior, leading to differences between the axial and transverse compression experienced at the UCNP surface, and thus the development of shear stress. Notably, this relationship is also nonlinear. The tenfold difference between confocal-AFM-measured force sensitivity and the force sensitivity approximated from DAC-measured pressure sensitivity via Eq. 2 should be viewed in the context of these simplistic assumptions.

Raman spectra (HR Evolution, Horiba Labram; 600 gr/mm grating; 785 nm excitation) of polystyrene microbeads lacking UCNPs as a function of pressure were taken from inside a DAC (BX-90, One20DAC, Almax EasyLab). Three 60s spectra were acquired and averaged for each pressure. The polystyrene signal overlaps partially with that of the pressure transmitting medium, silicone oil, for which the ambient pressure spectrum is shown in Extended Data Fig. 4b. Because it was challenging to distinguish between the two aliphatic peaks in polystyrene and the lowe- energy aliphatic peak in silicone oil from at all pressures, the three were fitted to a single Gaussian (purple, labeled P-Al+S-Al_1_ in Extended Data Fig. 4b). The other two peaks correspond to the higher-energy aliphatic peak of silicone oil (red, S-Al_2_) and the aromatic peak of polystyrene (yellow, P-Ar). Polystyrene microbeads were prepared using the same procedure used to prepare microgauges, but without UCNPs. The wide opening angle (120°) and short working distance (10 mm) simplify optical measurements. The DAC was prepared and operated as described above, with a 150 μm diameter gasket hole and 300 μm diameter diamond culets.

### *Caenorhabditis elegans* culturing and microgauge feeding

Animals were age-synchronized by first isolating eggs from gravid adults via hypochlorite treatment (200 μL household bleach and 20 μL of 5M potassium hydroxide added to gravid adult worms suspended in 1 mL water) and then by transferring eggs to 6 cm growth plates (nematode growth medium (NGM), 2% w/v agar) with food (*E. coli* OP50; optical density at 600 nm, 0.3) and incubated at 20°C. The culturing duration and feeding procedure varied depending on the application. For biocompatibility assays, worms were transferred after incubation for 69 hours to NGM plates seeded with 50 μL *E. coli* OP50, 250 μg SDS, and 100 μg microgauges and allowed to feed for three hours. Separate egg-laying plates with improved optical clarity were prepared in four-well microtiter plates containing 2.5% Gel-rite (RPI) medium (KPO_4_ (5mM), pH 6, supplemented with MgCl_2_ (1 mM) and CaCl_2_, and cholesterol (1:1,000 v/v from stock solution, 5 mg/mL in ethanol)), and each well was subsequently seeded with 50 μL *E.* coli OP50 as described previously (Supplementary Fig. 2).^58^ For correlated electrical-optical imaging, worms were transferred at hour 60 to NGM plates seeded with 15 μL *E. coli* OP50 and 100 μg microgauges and allowed to feed for 12 hours. Finally, to compare the ingestion of singly dispersed PMAO-wrapped UCNPs with ingestion of polymer-embedded UCNPs, worms were transferred at hour 69 to feed in M9 buffer without bacteria for one hour under gentle shaking. The buffer consisted of 800 μg microgauges (45 μg UCNPs, see Supplementary Fig. 3 for mass fraction determined with thermogravimetric analysis) or 45 μg PMAO-wrapped UCNPs. M9 (pH ~7) was prepared by mixing 3 g of KH_2_PO_4_ (Sigma-Aldrich), 6 g of Na_2_HPO_4_ (Sigma-Aldrich), 5 g of NaCl (Sigma-Aldrich), and 1L of ultrapure water. For the accumulation comparison, worms were fixed on agarose pads with a drop of 5 mM levamisole hydrochloride (TCI Chemicals) prior to imaging.

### Biocompatibility assays

To compare progeny among a cohort, two groups of eight worms were cultured and fed as described above, then transferred one at a time to egg-laying plates. Worms were transferred to a new well every 24 hours for a total of four days, and each well was imaged on a flatbed scanner (Perfection v600, Epson), modified as described,^58^ 48 hours later. To distinguish debris from mobile worms, reference images of each well were acquired five minutes later. Using FIJI, the progeny present in each well were counter by at least 2 experimenters blinded to each treatment, and the average value was used in each case (Supplementary Fig. 2). To compare pumping rate among a cohort, two groups of 15 worms were cultured and fed as described above.

### Electropharyngeogram (EPG) acquisition

EPGs were measured using a commercial microfluidics chip (ScreenChip30, InVivo Biosystems). The chip was prepared by flushing it with M9 containing serotonin (4.7 mM, Sigma-Aldrich, Cat. #H9523), to increase the pumping rate, as previously described.^59^ Synchronized young adult worms were collected from unseeded NGM growth plates in this buffer and injected into the staging area via a length of polyethylene tubing and a manual syringe. Worms were allowed to adapt to the immobilization channel for 60 s before EPG recording and imaging. The chip was positioned on the stage of an inverted microscope (Zeiss Axio Observer), and the position of the worm in the immobilization channel was verified by observation at low magnification (Zeiss 10x, 0.2 NA EC Epiplan). The applications NemAcquire and NemAnalysis (InVivo Biosystems) were used to collect the time course (500 Hz) and identify waveform features, respectively.

### UCL image acquisition *in vivo*

All UCL from microgauges within the pharyngeal lumen was captured in dual color channels on a digital camera (Orca Flash 4.0, Hamamatsu) fitted with a W-View Gemini Beam Splitter fitted with a 568 nm dichroic (Hamamatsu). Prior to imaging, channel images were aligned by adjusting the internal focusing lenses of the Gemini beam splitter. Furthermore, reference images of a fiduciary slide were used in postprocessing to correct translational misalignments at the pixel level (Supplementary Fig. 14b). Widefield imaging was performed on an inverted microscope (Zeiss Axio Observer) at 50x magnification (0.55 NA EC Epiplan NEOFLUAR objective) under an excitation power density of 13 kW/cm^2^ (MDL-N-980–8W). The camera was controlled with the manufacturer’s software, HC Image Live. Single-channel UCL images were recorded at 66 fps, and the full pixel array (2,048×1,024, no binning) was used. For correlated electrical and dual-channel optical video recordings, the camera was operated in external start trigger mode at 50 fps using a 512×128 pixel subset per channel and 2x binning. In this mode, the camera was set to begin acquisition after receiving a 3.3 V trigger pulse from an Arduino Leonardo. This Arduino also initiated the EPG acquisition via a near-synchronous mouse click in the NemAcquire software. This method results in an average lag of 64 ms (±18 ms) between the onset of acquisition in the two data streams. This corresponds to an average uncertainty in the alignment of the data streams equivalent to a single image frame (Supplementary Fig. 13). The maximum offset was two frames in either direction of the mean. Between one and five videos were acquired for each worm before the animal was ejected from the channel. Images of the empty channel collected under the same exposure conditions were used for background subtraction (Supplementary Fig. 14a). Average pixel intensities in the terminal bulb regions of the red and green channels were extracted with built-in morphological image processing functions in MATLAB (Supplementary Fig. 14c-e). A DC offset was applied to the voltage trace in each event (Supplementary Fig. 15). See Supplementary Fig. 16 and the Supplementary Information Section 7 for exclusion criteria applied to the optical and electrical datasets.

## Extended Data

**Extended Data Fig. 1:**
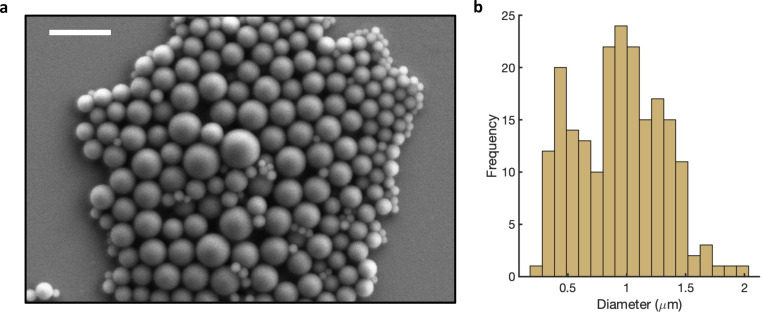
Microgauge size measured via SEM **a)** A SEM micrograph of a monolayer of uncoated microgauges on silicon. Scale bar, 3 μm. **b)** The distribution of N = 202 microgauge diameters (mean ± s.d. = 935 ± 367 nm), repeated for clarity from Fig. 1b).

**Extended Data Fig. 2:**
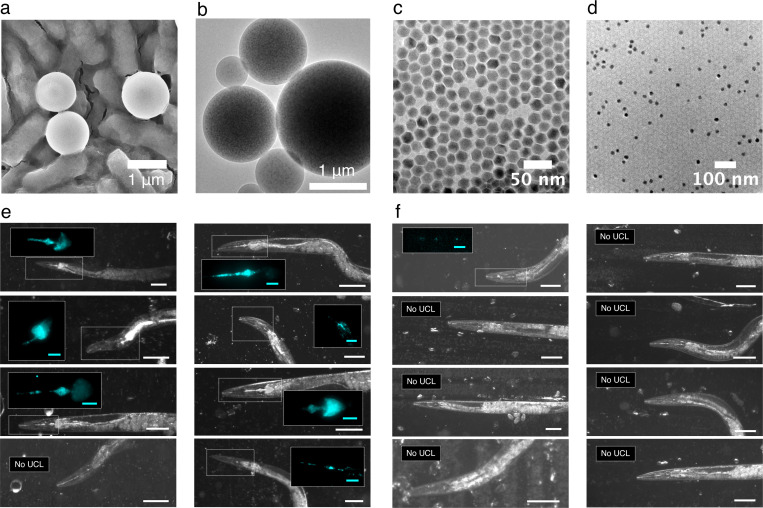
The effect of sensor size on pharyngeal accumulation efficiency **a)** An SEM micrograph of microgauges on *E. coli* for size comparison. **b)** A TEM micrograph of a microgauge. TEM micrographs of core@shell NaY_0.8_Yb_0.18_Er_0.02_F_4_@NaYF_4_ UCNPs (**c**) before and (**d**) after wrapping with PMAO. Wrapped nanoparticles were dropcast from water. A series of bright field images of levamisole-treated worms after one hour incubation with (**e**) microgauges or (**f**) singly dispersed PMAO-wrapped UCNPs. Scale bars are each 100 μm. False-colored UCL images (cyan) of the boxed pharyngeal regions are displayed in the inset of each picture, when applicable. Inset scale bars are each 20 μm each. The anterior of each animal is to the left.

**Extended Data Fig. 3:**
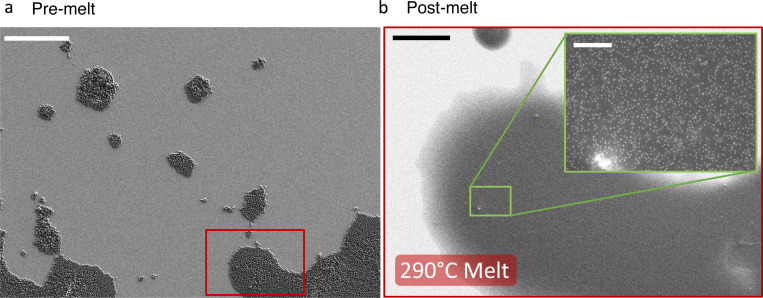
Microgauge monolayers before and after melting **a)** An SEM micrograph of microgauge monolayers on hydrophilized silicon. Scale bar, 50 μm. **b)** The same region after melting (30 minutes), with a higher resolution inset to show nanoparticles distributed evenly across the surface. Scale bars, 10 μm, 500 nm (inset). Clusters like the one shown in the bottom left of the inset were easily visible from confocal UCL maps and were avoided during the calibration.

**Extended Data Fig. 4:**
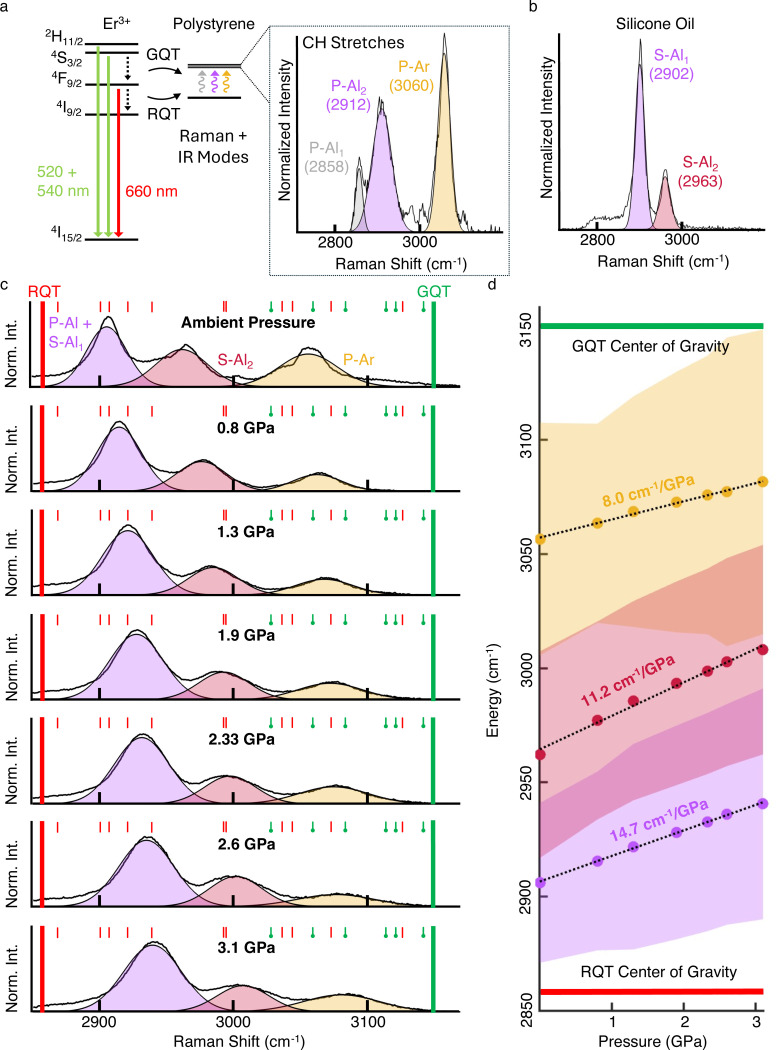
Er-Polystyrene energy overlap **a)** A simplified energy diagram of Er^3+^ that summarizes matrix quenching. Solid lines indicate radiative transitions, and dotted lines indicate non-radiative Energy Transfer (ET) to the aliphatic (gray and purple) and aromatic (yellow) C-H stretching modes on polystyrene. The corresponding Raman spectrum for polystyrene microbeads without nanoparticles at room temperature and ambient pressure (right). **b)** Raman spectrum for silicone oil (room temperature, ambient pressure). **c)** Raman spectra of polystyrene microbeads and silicone oil between ambient pressure (top) and 3.1 GPa (bottom). Fitted curves are filled in using the same color scheme as in panels (**a**) and (**b**). P-Al_1_, P-Al_2_ and S-Al_1_, too close to deconvolve at all pressures, are fitted to a single peak. Thick vertical lines indicate the centers of mass of the red quenching ^4^F_9/2_ → ^4^I_9/2_ (2858 cm^−1^) and green quenching ^4^S_3/2_ → ^4^F_9/2_ transitions (3150 cm^−1^), respectively (values obtained for LaF_3_ from Carnall et al.^60^). Smaller red and green (circular end cap) colored lines above the spectra indicate the transition energies between individual stark levels that are above and below the centers of mass, respectively. Values in cm^−1^ are as follows; RQT: 2871, 2904, 2912, 2924, 2941, 2992, 2994, 3038, 3046, 3075, 3128; GQT: 3030, 3059, 3083, 3112, 3112, 3120, 3141 (values obtained for LaF_3_ from Carnall et al.^60^). **d)** Peak energy (dots) and 2x standard deviations (colored area) of the fitted Gaussian. Dotted black lines are the respective linear fits of the peak energies with pressure.

**Extended Data Fig. 5:**
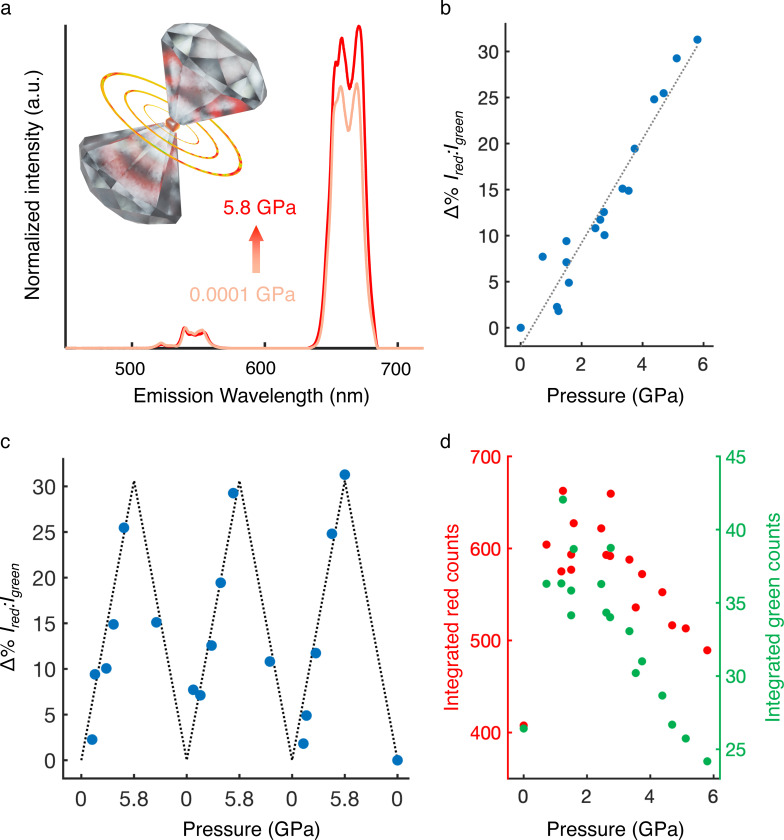
I_Red_:I_Green_ increases with (quasi)hydrostatic pressure **a)** Two spectra taken at either end of the pressure ramp showing the increase in relative red emission at high pressure. Both spectra are normalized to their respective green emission peaks. Inset, a schematic of the setup, wherein microgauges are compressed between two diamond culets. **b)** Ratiometric emission changes across three consecutive force loading-unloading cycles between ambient (0.0001 GPa) and high (5.8 GPa) pressure. The black line is the best fit line calculated in **c)**, where all ratiometric changes are plotted versus pressure regardless of loading or unloading status. **d)** The corresponding background corrected red (left axis, red) and green (right axis, green) UCL intensities as a function of pressure.

## Supplementary Material

Supplement 1

## Figures and Tables

**Figure 1:**
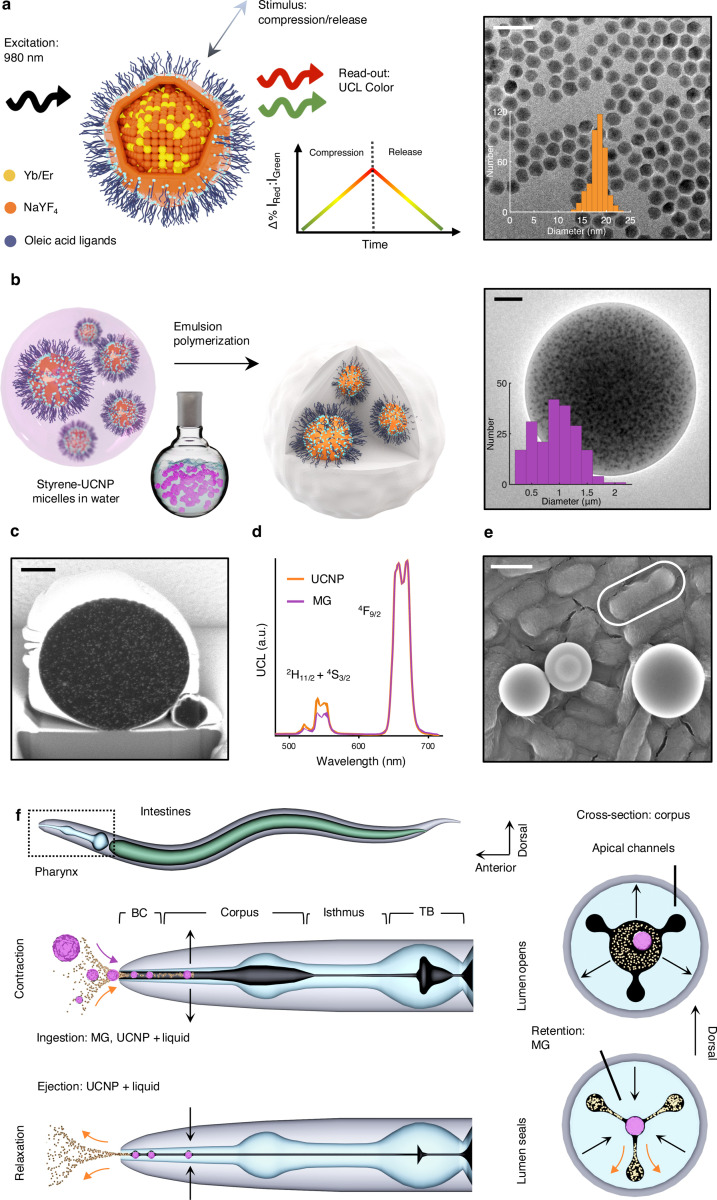
Synthesis of biocompatible microgauges compatible with passive ingestion **a**) Left: A schematic of a core@shell *α*-NaY_0.8_Yb_0.18_Er_0.02_F_4_@NaYF_4_ UCNP mechanosensor and its operation, in which UCL color (Δ%I_Red_:I_Green_) becomes redder during compression. Right: TEM image of core@shell UCNPs and a histogram of their diameters (18.2 ± 1.7 nm; mean ± s.d., *n* = 395). **b**) Left: A schematic of the emulsion polymerization scheme, wherein UCNP/styrene micelles undergo polymerization once heated. Right: A TEM image of a single microgauge with UCNPs visible and a histogram of microgauge diameters (935 ± 367 nm, *n =* 202). **c**) An SEM image of a single microgauge cross section after ion beam milling (see Supplementary Video 1 for all cross sections). Platinum deposition (outer white), polystyrene (black), and UCNPs (white flecks) are visible. **d**) *α*-NaY_0.8_Yb_0.18_Er_0.02_F_4_@NaYF_4_ UCL spectra (exc. 980 nm) before (orange) and after (purple) polystyrene encapsulation. **e**) An SEM image of microgauges on *E. coli* (outlined in white). **f**) A schematic of a *C. elegans* roundworm. Below is a magnified pharynx undergoing the contraction (top) and relaxation (bottom) phases of a pharyngeal pump. Right: procorpus cross sections showing the central lumen contracting open (top) and relaxing closed (bottom). The black arrows indicate the direction of muscle movement, and the orange and purple arrows indicate the direction of UCNP and microgauge movement, respectively. BC = buccal cavity, TB = terminal bulb. Scale bars are 50 nm (**a**), 200 nm (**b**), 500 nm (**c**), and 1 μm (**e**).

## Data Availability

Source data for Figs. 1–4 are available via the Stanford Digital Repositories (SDR) service at https://doi.org/10.25740/ff923hb3417. All other source data are available upon reasonable request.

## References

[1] Lockery S. R. A microfluidic device for whole-animal drug screening using electrophysiological measures in the nematode C. elegans. Lab Chip 12, 2211–2220 (2012). 22588281 10.1039/c2lc00001f PMC3372093

[2] Oh Y. An orange calcium-modulated bioluminescent indicator for non-invasive activity imaging. Nat. Chem. Biol. 15, 433–436 (2019). 30936501 10.1038/s41589-019-0256-z PMC6563924

[3] Tsutsui H., Higashijima S.-I., Miyawaki A. & Okamura Y. Visualizing voltage dynamics in zebrafish heart. J. Physiol. 588, 2017–2021 (2010). 20421282 10.1113/jphysiol.2010.189126 PMC2911208

[4] Eyckmans J., Boudou T., Yu X. & Chen C. S. A hitchhiker’s guide to mechanobiology. Dev. Cell 21, 35–47 (2011). 21763607 10.1016/j.devcel.2011.06.015 PMC3155761

[5] Mehlenbacher R. D., Kolbl R., Lay A. & Dionne J. A. Nanomaterials for in vivo imaging of mechanical forces and electrical fields. Nature Reviews Materials 3, 1–17 (2017).

[6] Yu L., Kim B. J. & Meng E. Chronically implanted pressure sensors: challenges and state of the field. Sensors 14, 20620–20644 (2014). 25365461 10.3390/s141120620 PMC4279503

[7] Wuytack E. Y., Diels A. M. J. & Michiels C. W. Bacterial inactivation by high-pressure homogenisation and high hydrostatic pressure. Int. J. Food Microbiol. 77, 205–212 (2002). 12160080 10.1016/s0168-1605(02)00054-5

[8] Donsì F., Ferrari G., Lenza E. & Maresca P. Main factors regulating microbial inactivation by high-pressure homogenization: Operating parameters and scale of operation. Chem. Eng. Sci. 64, 520–532 (2009).

[9] Chumpitazi B. & Nurko S. Pediatric gastrointestinal motility disorders: challenges and a clinical update. Gastroenterol. Hepatol. 4, 140–148 (2008). PMC3088841 21904491

[10] Dorsher P. T. & McIntosh P. M. Neurogenic bladder. 2012, (2012). 10.1155/2012/816274 PMC3287034 22400020

[11] Shah M., Akar F. G. & Tomaselli G. F. Molecular basis of arrhythmias. Circulation 112, 2517–2529 (2005). 16230503 10.1161/CIRCULATIONAHA.104.494476

[12] Krieg M. Atomic force microscopy-based mechanobiology. Nature Reviews Physics 1, 41–57 (2018).

[13] Fox M. R. & Bredenoord A. J. Oesophageal high-resolution manometry: moving from research into clinical practice. Gut 57, 405–423 (2008). 17895358 10.1136/gut.2007.127993

[14] Chen J.-H. Intraluminal pressure patterns in the human colon assessed by high-resolution manometry. Sci. Rep. 7, (2017). 10.1038/srep41436 PMC5316981 28216670

[15] Meng F., Suchyna T. M. & Sachs F. A fluorescence energy transfer-based mechanical stress sensor for specific proteins in situ. FEBS J. 275, 3072–3087 (2008). 18479457 10.1111/j.1742-4658.2008.06461.x PMC2396198

[16] Borghi N. E-cadherin is under constitutive actomyosin-generated tension that is increased at cell–cell contacts upon externally applied stretch. Proceedings of the National Academy of Sciences 109, 12568–12573 (2012). 10.1073/pnas.1204390109 PMC3411997 22802638

[17] Grashoff C. Measuring mechanical tension across vinculin reveals regulation of focal adhesion dynamics. Nature 466, 263–266 (2010). 20613844 10.1038/nature09198 PMC2901888

[18] Krieg M., Dunn A. R. & Goodman M. B. Mechanical control of the sense of touch by β-spectrin. Nat. Cell Biol. 16, 224–233 (2014). 24561618 10.1038/ncb2915 PMC4046587

[19] Xiong L., Yang T., Yang Y., Xu C. & Li F. Long-term in vivo biodistribution imaging and toxicity of polyacrylic acid-coated upconversion nanophosphors. Biomaterials 31, 7078–7085 (2010). 20619791 10.1016/j.biomaterials.2010.05.065

[20] Zhou J.-C. Bioimaging and toxicity assessments of near-infrared upconversion luminescent NaYF4:Yb,Tm nanocrystals. Biomaterials 32, 9059–9067 (2011). 21880365 10.1016/j.biomaterials.2011.08.038

[21] Lay A. Optically Robust and Biocompatible Mechanosensitive Upconverting Nanoparticles. ACS Cent Sci 5, 1211–1222 (2019). 31403071 10.1021/acscentsci.9b00300 PMC6661856

[22] Chen S. Near-infrared deep brain stimulation via upconversion nanoparticle–mediated optogenetics. Science 359, 679–684 (2018). 29439241 10.1126/science.aaq1144

[23] Xu J. Highly Emissive Dye-Sensitized Upconversion Nanostructure for Dual-Photosensitizer Photodynamic Therapy and Bioimaging. ACS Nano 11, 4133–4144 (2017). 28320205 10.1021/acsnano.7b00944

[24] Chatterjee D. K., Rufaihah A. J. & Zhang Y. Upconversion fluorescence imaging of cells and small animals using lanthanide doped nanocrystals. Biomaterials 29, 937–943 (2008). 18061257 10.1016/j.biomaterials.2007.10.051

[25] Haase M. & Schäfer H. Upconverting nanoparticles. Angew. Chem. Int. Ed Engl. 50, 5808–5829 (2011). 21626614 10.1002/anie.201005159

[26] Lay A. Upconverting Nanoparticles as Optical Sensors of Nano- to Micro-Newton Forces. Nano Lett. 17, 4172–4177 (2017). 28608687 10.1021/acs.nanolett.7b00963 PMC6589185

[27] McLellan C. A. Engineering Bright and Mechanosensitive Alkaline-Earth Rare-Earth Upconverting Nanoparticles. J. Phys. Chem. Lett. 13, 1547–1553 (2022). 35133831 10.1021/acs.jpclett.1c03841 PMC9587901

[28] Lay A. Bright, Mechanosensitive Upconversion with Cubic-Phase Heteroepitaxial Core–Shell Nanoparticles. Nano Lett. 18, 4454–4459 (2018). 29927609 10.1021/acs.nanolett.8b01535 PMC6613353

[29] Kwok T. C. Y. A small-molecule screen in C. elegans yields a new calcium channel antagonist. Nature 441, 91–95 (2006). 16672971 10.1038/nature04657

[30] Schüler C., Fischer E., Shaltiel L., Steuer Costa W. & Gottschalk A. Arrhythmogenic effects of mutated L-type Ca 2+-channels on an optogenetically paced muscular pump in Caenorhabditis elegans. Sci. Rep. 5, (2015). 10.1038/srep14427 PMC4585839 26399900

[31] Lee R. Y. N., Lobel L., Hengartner M., Horvitz H. R. & Avery L. Mutations in the α1 subunit of an L-type voltage-activated Ca2+ channel cause myotonia in Caenorhabditis elegans. EMBO J. 16, 6066–6076 (1997). 9321386 10.1093/emboj/16.20.6066 PMC1326290

[32] Corsi A. K., Wightman B. & Chalfie M. A Transparent Window into Biology: A Primer on Caenorhabditis elegans. Genetics 200, 387–407 (2015). 26088431 10.1534/genetics.115.176099 PMC4492366

[33] Fischer S., Bronstein N. D., Swabeck J. K., Chan E. M. & Alivisatos A. P. Precise Tuning of Surface Quenching for Luminescence Enhancement in Core–Shell Lanthanide-Doped Nanocrystals. Nano Lett. 16, 7241–7247 (2016). 27726405 10.1021/acs.nanolett.6b03683

[34] Lahtinen S. Disintegration of Hexagonal NaYF4:Yb3+,Er3+ Upconverting Nanoparticles in Aqueous Media: The Role of Fluoride in Solubility Equilibrium. J. Phys. Chem. C 121, 656–665 (2017).

[35] Kraft M., Würth C., Muhr V., Hirsch T. & Resch-Genger U. Particle-size-dependent upconversion luminescence of NaYF4: Yb, Er nanoparticles in organic solvents and water at different excitation power densities. Nano Res. 11, 6360–6374 (2018).

[36] Casar J. R., McLellan C. A., Siefe C. & Dionne J. A. Lanthanide-Based Nanosensors: Refining Nanoparticle Responsiveness for Single Particle Imaging of Stimuli. ACS Photonics 8, 3–17 (2021). 34307765 10.1021/acsphotonics.0c00894 PMC8297747

[37] Qian H., Li Z. & Zhang Y. Multicolor polystyrene nanospheres tagged with up-conversion fluorescent nanocrystals. Nanotechnology 19, (2008). 10.1088/0957-4484/19/25/255601 21828653

[38] Fang-Yen C., Avery L. & Samuel A. D. T. Two size-selective mechanisms specifically trap bacteria-sized food particles in Caenorhabditis elegans. Proc. Natl. Acad. Sci. U. S. A. 106, 20093–20096 (2009). 19903886 10.1073/pnas.0904036106 PMC2785297

[39] Brenner I. R., Raizen D. M. & Fang-Yen C. Pharyngeal timing and particle transport defects in Caenorhabditis elegans feeding mutants. J. Neurophysiol. 128, 302–309 (2022). 35730757 10.1152/jn.00444.2021

[40] Sparacio A. P., Trojanowski N. F., Snetselaar K., Nelson M. D. & Raizen D. M. Teething during sleep: Ultrastructural analysis of pharyngeal muscle and cuticular grinder during the molt in Caenorhabditis elegans. PLoS One 15, (2020). 10.1371/journal.pone.0233059 PMC7239488 32433687

[41] Hodgkin J. & Barnes T. M. More is not better: brood size and population growth in a self-fertilizing nematode. Proc. Biol. Sci. 246, 19–24 (1991). 1684664 10.1098/rspb.1991.0119

[42] Raizen D. M. & Avery L. Electrical activity and behavior in the pharynx of Caenorhabditis elegans. Neuron 12, 483–495 (1994). 8155316 10.1016/0896-6273(94)90207-0 PMC4460247

[43] Avery L. The genetics of feeding in Caenorhabditis elegans. Genetics 133, 897–917 (1993). 8462849 10.1093/genetics/133.4.897 PMC1205408

[44] Portal-Celhay C., Bradley E. R. & Blaser M. J. Control of intestinal bacterial proliferation in regulation of lifespan in Caenorhabditis elegans. BMC Microbiol. 12, 49 (2012). 22452899 10.1186/1471-2180-12-49 PMC3342110

[45] Kumar S. Lifespan Extension in C. elegans Caused by Bacterial Colonization of the Intestine and Subsequent Activation of an Innate Immune Response. Dev. Cell 49, 100–117.e6 (2019). 30965033 10.1016/j.devcel.2019.03.010 PMC6946027

[46] Vega N. M. & Gore J. Stochastic assembly produces heterogeneous communities in the Caenorhabditis elegans intestine. PLoS Biol. 15, e2000633 (2017). 10.1371/journal.pbio.2000633 PMC5336226 28257456

[47] Johnson K. L. Contact Mechanics. (Cambridge University Press, 1987).

[48] Auer G. K. & Weibel D. B. Bacterial Cell Mechanics. Biochemistry 56, 3710–3724 (2017). 28666084 10.1021/acs.biochem.7b00346 PMC6260806

[49] Johnson K. L., Kendall K. & Roberts A. D. Surface energy and the contact of elastic solids. Proc. R. Soc. Lond. 324, 301–313 (1971).

[50] Guruprasad T. S., Bhattacharya S. & Basu S. Size effect in microcompression of polystyrene micropillars. Polymer 98, 118–128 (2016).

[51] Herndon L. A. Stochastic and genetic factors influence tissue-specific decline in ageing C. elegans. Nature 419, 808–814 (2002). 12397350 10.1038/nature01135

[52] Kashyap L., Perera S. & Fisher A. L. Identification of novel genes involved in sarcopenia through RNAi screening in Caenorhabditis elegans. J. Gerontol. A Biol. Sci. Med. Sci. 67, 56–65 (2012). 21593014 10.1093/gerona/glr072 PMC3260486

[53] Jiang G., Pichaandi J., Johnson N. J. J., Burke R. D. & van Veggel F. C. J. M. An effective polymer cross-linking strategy to obtain stable dispersions of upconverting NaYF4 nanoparticles in buffers and biological growth media for biolabeling applications. Langmuir 28, 3239–3247 (2012). 22250577 10.1021/la204020m

[54] Schindelin J. Fiji: an open-source platform for biological-image analysis. Nat. Methods 9, 676–682 (2012). 22743772 10.1038/nmeth.2019 PMC3855844

[55] Schietinger S., Aichele T., Wang H.-Q., Nann T. & Benson O. Plasmon-enhanced upconversion in single NaYF4:Yb3+/Er3+ codoped nanocrystals. Nano Lett. 10, 134–138 (2010). 20020691 10.1021/nl903046r

[56] Mao H. K., Bell P. M., Shaner J. W. & Steinberg D. J. Specific volume measurements of Cu, Mo, Pd, and Ag and calibration of the ruby R1 fluorescence pressure gauge from 0.06 to 1 Mbar. J. Appl. Phys. 49, 3276–3283 (1978).

[57] Klotz S., Chervin J.-C., Munsch P. & Le Marchand G. Hydrostatic limits of 11 pressure transmitting media. J. Phys. D Appl. Phys. 42, 075413 (2009).

[58] Fryer E. A high-throughput behavioral screening platform for measuring chemotaxis by C. elegans. PLoS Biol. 22, (2024). 10.1371/journal.pbio.3002672 PMC11210793 38935621

[59] Horvitz H. R., Chalfie M., Trent C., Sulston J. E. & Evans P. D. Serotonin and octopamine in the nematode Caenorhabditis elegans. Science 216, 1012–1014 (1982). 6805073 10.1126/science.6805073

[60] Carnall W. T., Crosswhite H. & Crosswhite H. M. Energy Level Structure and Transition Probabilities in the Spectra of the Trivalent Lanthanides in LaF₃. http://dx.doi.org/10.2172/6417825 (1978) doi:10.2172/6417825.

