# Supplementary material for: Upconverting microgauges reveal intraluminal force dynamics in vivo
Source: ArXiv. 2025 Oct 9:arXiv:2510.07657v1. Preprint. [Version 1] (PMC12632686)
Supplement: Supplement 1 [file NIHPP2510.07657v1-supplement-1.pdf]

Supplementary Information: Upconverting microgauges reveal intraluminal force dynamics *in vivo*

Jason R. Casar[7*], Claire A. McLellan[7], Cindy Shi[7], Ariel Stiber[7], Alice Lay[1], Chris Siefe[7], Abhinav Parakh[2,7], Malaya Gaerlan[3,7], Wendy Gu[4], Miriam B. Goodman[5*], Jennifer A. Dionne[6,7,8*]

[1]Department of Applied Physics, Stanford University, Stanford CA, 94305, USA

[2]Materials Engineering Division, Lawrence Livermore National Laboratory, Livermore CA, 94550, USA

[3]Department of Biology, Stanford University, Stanford CA, 94305, USA

[4]Department of Mechanical Engineering, Stanford University, Stanford CA, 94305, USA

[5]Department of Molecular and Cellular Physiology, Stanford University, Stanford CA, 94305, USA

[6]Department of Radiology, Stanford University, Stanford CA, 94305, USA

[7]Department of Materials Science and Engineering, Stanford University, Stanford CA, 94305, USA

[8]Chan Zuckerberg Biohub, San Francisco, San Francisco, CA 94158

*Address correspondence to: jcasar@stanford.edu; mbgoodman@stanford.edu; jdionne@stanford.edu

Contents

Section 1: C. elegans toxicity **3**
Laser heating assay 3
Progeny Counting 3
Section 2: Thermogravimetric analysis **4**
Section 3: Confocal microscopy **4**
Confocal optical train 4
Co-alignment for simultaneous imaging and spectroscopy 6
Section 4: Nanoindentation **6**
Compressive modulus characterization 6
Contact pressure estimation 8
Plastic deformation tests 10
Section 5: Microgauge pressure sensitivity mechanisms **11**
Er-Er energy transfer 12
Er-Polystyrene energy transfer 16
Radiative rate enhancement 19
(Quasi)hydrostatic pressure dependence of red and green UCL intensities 21
Section 6: Diamond Anvil Cell Preparation and Imaging **23**
Section 7: Electrical and optical measurement of pharyngeal function **24**
Signal synchronization: Trigger setup and lag 24
Lag estimation 25
Event-triggered averages 26
Optical data processing 26
EPG data processing 27
Event-triggered averaging of optical and electrical signals 29
Exclusion criteria 29
Section 8: Supplementary Figures **33**

Section 1: *C. elegans* toxicity

Laser heating assay

We adopted established bioassays for heat stress in *C. elegans* to evaluate the possibility that NIR illumination itself might be toxic to living animals. This assay relies on transgenic animals (TJ375 *gpIs1*[hsp-16.2p::GFP]) expressing GFP under the control of the promoter of the *hsp-16* heat-shock protein gene. This gene expression reporter generates visible fluorescence in the muscles of the pharynx 15-18 hours after heating to 35°C or above for 1-2 hours.[1] (*C. elegans* are healthy at temperatures between 15 and 26°C.) Replicating this finding, GFP fluorescence was stronger 16 hours after 2 hours at 37°C than 2 hours at 20°C (data not shown). Having established the utility of this assay as a heat-stress reporter, we analyzed young (day 1) adult worms for GFP fluorescence 16 hours after one of three conditions: 1) Control temperature (20°C), 2 hours; 2) Noxious heat (37°C), 2 hours, and 3) NIR (980nm, 13 kW/cm^2) laser irradiation, 1 minute. The last condition was performed on worms immobilized in the microfluidics devices used for EPGs and mechanical imaging. Control and noxious temperatures were achieved by placing animals in temperature-controlled incubators. As shown in SI Fig. 1, GFP expression was strongly upregulated in animals treated at 37°C for 2 hours. Based upon quantification of GFP expression in the head, the response to NIR irradiation was an order of magnitude lower. Treatment at 20° for 2 hours produced no detectable GFP expression. Thus, NIR laser irradiation seems to produce a mild heat stress response.

Progeny Counting

The total brood size of adult worms is a useful window into toxicity. The general approach is to place single animals in one well of a 4-well plate, transferring them daily to a new egg-laying well for 3 days, and then to count the number of progeny produced by each parent worm. Assay plates were imaged

twice on a flat-bed scanner, and progeny were counted by hand by an experimenter unaware of the treatment condition (SI Fig. 2). As seen in the zoomed-in green box, the size, shape, and contrast of the worms make them clearly distinguishable from bacterial food and debris. The second scan, taken 5' after the first one, further helped to differentiate debris or scratches from mobile worms.

Section 2: Thermogravimetric analysis

Using fluorescence as a metric to compare the pharyngeal accumulation of PMAO-wrapped UCNPs (vs. microgauges) requires that we deliver an equal nanoparticle mass to animals in both conditions. We determined the mass fraction of $NaY_{0.8}Yb_{0.18}Er_{0.02}F_4@NaYF_4$ UCNPs in microgauges via thermogravimetric analysis (TGA, TA Instrument Q500). Samples were prepared as follows: 5.04 mg of sample was transferred to a flame-dried pan, heated at a rate of 20°C/min to 110°C, and held at that temperature 20 minutes to remove water. Sample mass was recorded as the pan was heated from 110°C to 550°C at a rate of 10°C/min under a nitrogen flow of 100mL/min (SI Fig. 3). To estimate how much of the remaining mass was due to residual, unoxidized carbon, we also tested polystyrene microspheres. This control was synthesized using an identical procedure (without UCNPs) and ramped under the same test conditions. The difference in residual mass was calculated to be 5.7%.

Section 3: Confocal microscopy

Confocal optical train

A schematic of the entire optical train is provided in SI Fig. 4. All components are mounted in a vibration isolation enclosure except those depicted in the inset, which are mounted on an external floating optical table. Except for the microscope's internal tube lens, all lenses are achromatic doublets with the

appropriate anti-reflective coating (Thorlabs). Any unlabeled 45° lines represent silvered mirrors. We operated the 980 nm laser (Coherent OBIS) at 100 mW continuously. We measure a beam diameter of 0.7 mm exiting the laser and 1.75 mm entering the collimator (d) downstream of the lens pair. The collimator focuses the excitation onto a single mode fiber having a mode field diameter (MFD) of 5.3 μm (Thorlabs, P1-980A-FC-5) in order to clean the beam profile. Inside the enclosure, the beam profile is shaped by another lens pair. From this point, it passes through a 925-nm dichroic filter (FF01-Di01) to a Newport fast steering mirror (FSM-300-02). The FSM rasters the excitation beam across the sample (and also descans the UCL emission onto a common axis in the collection path). The addition of another lens pair allows us to expand the beam further to fill the back focal plane of the objective, and thus enhance spatial resolution. The objective is a cover glass (0.17 mm) corrected, 40x 0.95 NA Plan-Apochromat Zeiss objective with a back aperture diameter of 7.6 mm. We measure an overfill of 10 mm. The lateral resolution measured from the two channels is consistent (1.02 μm for transmission and 1.03 μm for reflection). The collection path deviates from the excitation path at the dichroic filter. We then filter all residual excitation light from the path with two shortpass filters (m,n). The collection path culminates in a 50:50 beamsplitter cube (Thorlabs, CCM1-BS013/M) that partitions the beam into the imaging and spectroscopy channels. Here, two identical single-mode fiber-collimator pairs function as fixed pinholes. Small thermal fluctuations of the stage can result in tens to hundreds of nanometers of drift, which can affect the power delivered to the sample (axial drift) or the force experienced by the excited region of the film (lateral drift). We minimize thermal drift by holding the AFM head at 26°C (± 0.02°C); it takes approximately 12 hours for the temperature controller to achieve this equilibrium inside the sealed vibration isolation chamber.

Co-alignment for simultaneous imaging and spectroscopy

To guarantee that thermal drift did not affect the real-time coincidence between the confocal excitation spot and the center of the AFM tip-sample contact, we built two separate collection channels into the confocal optical train. The first was coupled to a spectrometer for mechano-optical calibration, as described previously. The second was coupled to a single photon counting device (SPCD) to monitor UCL counts during a spectral acquisition, and generate images between loading-unloading cycles. Near-ideal channel co-alignment was confirmed by overlaying diffraction-limited images of single nanoparticles. The average X and Y emission profiles of ten images taken over the course of 10 minutes on each channel were fit to a 2D Gaussian (SI Fig. 5). Finally, we determined the initial coincidence between the excitation spot and contact area for each film location by indenting the tip to our reference force point (0.4 μN) and observing the change in emission profile of the film. The center of the excitation spot is positioned at the center of the spherical contact area (SI Fig. 6).

Section 4: Nanoindentation

Compressive modulus characterization

We generated force-indentation curves from 50 microgauges according to methods described in the main text. We then fit each approach curve to a Johnson-Kendall-Roberts (JKR) model using a gradient descent method. A JKR model was chosen to account for the adhesive forces that produced a distinct "jump-to-contact" in the approach curves. From this analysis, we calculated an average modulus of 740 MPa with a standard deviation of 410 MPa (SI Fig. 7), which is within the range reported for microscale polystyrene in similar AFM indentation studies.[2–4] We probed microgauges between 0.18 and 2 μm in diameter, and did not observe any significant size (volume) dependence in the measured moduli (SI Fig. 7c). For small indentations, the parabolic tip used can be approximated as a sphere with radius $R_{tip} = 30\,nm$. We extracted radii for the microgauges from their accompanying height maps, $R_{mg} =$

$AFM\ Height/2$. Finally, we assumed that the Poisson's ratio of the microgauges was similar to that of polystyrene, $\nu_{mg} = 0.265$.

The assumed analytical form for indentation, $\boldsymbol{\delta}$, according to the JKR model[5] is:

$$\hat{\delta} = \frac{F_J^{\frac{2}{3}} - \frac{4}{3}(F_J F_{ad})^{\frac{1}{3}}}{(R_{eff}K^2)^{\frac{1}{3}}} \qquad \text{SI Eq.5}$$

where,

$$R_{eff} = \frac{1}{\frac{1}{R_{mg}} + \frac{1}{R_{tip}}}, \qquad \text{SI Eq. 6}$$

$$F_J = (\sqrt{F_{ad}} + \sqrt{F_{ad} + F})^2, \quad \text{and} \qquad \text{SI Eq. 7}$$

$$K = \frac{\frac{4}{3}E_{mg}}{1 - \nu_{mg}^2} \qquad \text{SI Eq. 8}$$

We estimated the adhesive force, F_{ad}, from the absolute value of the minimum of the baseline corrected approach curve following jump-to-contact. The exact onset of indentation, δ_0, is not easily distinguishable in force-indentation curves of adhesive, deformable samples such as the ones tested here. However, within the JKR model, there is an analytical dependence between δ_0 and an adhesive force such that $\delta(F) = \delta_0$ when $F_J(F) = 2.37F_{ad}$. Although K could more accurately be written as, $K_{eff} = \frac{1}{\frac{1-\nu_{mg}^2}{\frac{4}{3}E_{mg}} + \frac{1-\nu_{tip}^2}{\frac{4}{3}E_{tip}}}$, K is a good approximation when $E_{tip} \gg E_{mg}$. This is the case for our silicon tip (E_{tip}= 200 GPa). A sum of squared residuals loss function, $\sum(\hat{\delta} - \delta)^2$, was used to converge on an estimate of the fit parameter, *K*. From trial and error, we found that with a learning rate of $5 * 10^{13}$ we could achieve convergence to 10^{-5} GPa for all samples in under 10,000 iterations. $E_{mg}$was then extracted from the K value corresponding to the optimal fit according to SI Eq. 8. SI Fig. 7 shows a sample fit performed with this method and all 50 of the E_{mg} values obtained from this fitting procedure. The linear least squares fit of compressive modulus versus volume has an unconvincing R^2 of 0.44 (SI Fig. 7c). Thus, we do not

expect size to be significantly correlated to mechano-optical responsiveness.

Contact pressure estimation

Having an estimate of the compressive modulus of the microgauge material allows us to estimate the maximal Hertzian contact stresses experienced by the microgauge film under various loading conditions, and compare it to the compressive yield stress of microscale polystyrene.[6] For a spherical indenter of radius, R_{tip}, the maximum stress experienced by the sample for a given uniaxial compressive load, P, is given by[7]

$$\sigma_{max} = \frac{\sqrt[3]{6PE_{eff}^{\,2}}}{\sqrt[3]{\pi^3 R_{eff}^2}} \quad \text{SI Eq. 9}$$

And the mean contact stress is ⅔ this value.[7] In this case, we are assuming that the microgauge film being indented is flat with an infinite radius, and thus $R_{eff} \approx R_{tip}$, and that both the silica and silicon indenters are significantly stiffer than the film, $\frac{1}{E_{eff}} \approx \frac{1-\upsilon_{mg}^{\,2}}{E_{mg}} \approx \frac{1}{800\ MPa}$. Once again, we make the assumption that the indentation depth of the rounded parabolic indenter is small relative to its radius of curvature, so we can approximate it as a spherical indenter. Employing SI Eq. 9, we estimate that the maximal contact stress used in the mechano-optical characterization with the spherical silica tip is σ_{max}= 42 MPa. We employ this same analytical form to estimate the maximum contact stress experienced by the 1) microgauges and 2) *E. coli* within the grinder. To our knowledge, there is no experimentally derived stiffness for the grinder cuticle, so we estimate that it is similar to that of chitinous structures within animals that have been previously characterized with nanoindentation. These studies report average fitted moduli in the range of just under one GPa to over ten GPa.[8–11] We recognize that many factors affecting stiffness (such as the relative fraction of chitin, the nano-to-micrometer scale structural organization of chitin alone and in conjunction with other components, and the structure's porosity) are likely to differ

between structures. However, because this range reflects structures having a variety of functions derived from an array of different species, we believe it is likely to be representative of the grinder cuticle as well. Based on the assumption that the cuticle stiffness is ~1 GPa and has a Poisson's ratio similar to that of polystyrene, the effective modulus is $E_{eff} \approx 410\ MPa$. Depending what part of the lumen is in contact during compression, the contact area is limited by either the average microgauge diameter or the width of the grinder's individual teeth (~200-500 nm, 375 nm average).[12] These assumptions yield average Hertzian contact stresses for 15.7 μN of 84 MPa and 155 MPa, respectively.

As mentioned in the main text, high pressure homogenizers operating under different conditions between about 50 and 200 MPa[13,14] can achieve the same thousand-fold *E. coli* inactivation rate measured by Vega and Gore (2017) *in vivo*.[16] The pressure-to-force conversion we employ to estimate a corresponding force range relies on estimates of the geometry and stiffness of the cuticle (discussed above) as well as *E. coli*, which fall in the range of tens of MPa to a few hundred MPa.[15] For simplicity, we assume that the bacteria is oriented with its long dimension normal to the grinder cuticle, and can thus be modeled as a 500 nm diameter sphere with a compressive modulus of 50 MPa. An average hertzian contact stress of 50 (or 250) MPa would require a compressive force of 4.2 (or 270) μN.

Plastic deformation tests

We sought to confirm that the mechano-optical calibrations were performed within the elastic regime of the microgauge film. To do so, we replicated the same three-cycle loading conditions on a microgauge film sample and used scanning electron microscopy to visually compare the surface integrity before and after the indentation. A 30-second dwell time was used for each force actuation, and the sample was imaged without any conductive deposition layer to prevent interference with the compressive testing. No scarring or divotting was observed in the sample (SI Fig. 8). To confirm that it was possible to observe scarring with stresses exceeding the predicted yield strength, we applied three different forces, each at a

different film location, with a sharper 30 nm radius silicon tip (SI Fig. 9). Although it exceeded the estimated yield stress of 100 MPa by a large margin, the first force (0.5 nN, σ_{max}= 400 MPa) did not produce plastic deformation. The latter two (1 nN, σ_{max}= 510 MPa; 2.5 nN, σ_{max}= 700 MPa) did.

Section 5: Microgauge pressure sensitivity mechanisms

The trend towards redder Er^{3+} UCL color at high pressure is consistently observed in co-Yb-Er-doped nanoparticles.[17–19] This raises the questions: What material changes are taking place at high pressure, and what transitions within the UC network are impacted by these changes? The energy transfer (ET) network[20] that results in red and green emission from near-infrared excitation is complex, with a number of photon- and phonon-mediated processes. It is logical to expect that pressure would impact donor-acceptor pair separations, lattice or matrix phonon energies, index of refraction and local symmetry. Each of these could in turn impact any number of steps along the red and green UC pathways, including multiphonon relaxation (MPR), energy transfer upconversion (ETU), cross relaxation (CR), energy migration, and radiative emission. ED Fig. 4d, demonstrates that the rate of green UCL loss with pressure (33%) is faster than the rate of red UCL loss (20%) between 2.75 and 5.8 GPa, suggesting that differential loss mechanisms could contribute to observed color changes. This discussion will highlight two mechanisms that, if consequential, would produce a qualitatively similar trend, Er^{3+}-ion cross relaxation and matrix quenching.

Er-ion cross relaxation

Lattice strain will decrease donor-acceptor pair separation, a critical parameter that is known to enhance ET transitions that populate the red and green emitting levels but also increase the cross relaxation and energy-migration-to-surface processes that depopulate them.[20–22] Several studies suggest that cross

relaxation is an efficient mechanism for quenching green emission in Er-doped UCNPs. For example, Rabouw et al. showed that increasing erbium concentration from 0.1% to 2% enhances green quenching rates in β-$NaYF_4$ by three-fold, but has a relatively minor effect on red quenching rates.[23] The green quenching ET transitions Er ($^2H_{11/2}$→$^4I_{9/2}$): Er ($^4I_{15/2}$→$^4I_{13/2}$) and Er ($^2H_{11/2}$→$^4I_{13/2}$): Er ($^4I_{15/2}$→$^4I_{9/2}$) have an energy mismatch of 245 and 215 cm^{-1}, respectively. Compare this to the 2248 cm^{-1} mismatch of the Er ($^4F_{9/2}$→$^4I_{13/2}$): Er ($^4I_{15/2}$→$^4I_{13/2}$) transition, which is the smallest Er-Er cross relaxation process that quenches red emission to an acceptor in the ground state.[24] In SI Fig 12 we compare the expected nearest Er^{3+} acceptor (NEA) separation, $\hat{R}_{Er_i-NEA}$, for each potential Er^{3+} donor site in a cubic 10.9 nm diameter $NaYF_4$ core to the critical Förster radius for Er^{3+}-Er^{3+} cross relaxation calculated by Rabouw et al. (R_0 = 0.92 nm). In this analysis we assume an independent and homogenous probability, P = 0.01, that FCC acceptor sites are occupied by Er^{3+}, the lattice parameter is 5.51 Å,[17] the compressive modulus of the linear, isotropically elastic lattice is E = 272 GPa,[18] and the effects of unequal bond lengths are negligible. When the lattice is compressed hydrostatically from atmospheric pressure to 5.8 GPa, the proportion of donor sites with $\hat{R}_{Er_i-NEA}$ less than R_0 increases from 52% to 64% (SI Fig. 12 inset). This implies that, on average, more donor sites excited to the green emitting state would experience a cross relaxation rate that "outpaces" radiative emission. Er^{3+}-Yb^{3+} cross relaxation is another efficient green quenching mechanism in cubic Y_2O_3.[25] Dong et al. suggest that proximity induced enhancement of a Er^{3+}-Yb^{3+} cross relaxation-mediated green-to-red population pathway in non-stoichiometric, synthetically contracted α-$NaYF_4$ contributed to a fifty-fold I_{Red}:I_{Green} enhancement.[26] Furthermore, if we assume the ET rate from an excited Erbium donor has a FRET-type separation dependence, $W = \frac{C}{R^6}$, then the maximum pressure employed in our UCL DAC experiment (5.8 GPa) enhances the ET rate by 13.8%. These data suggest that cross relaxation is a mechanism which disproportionately depopulates the green emitting manifold of Er^{3+} donors, and which can be enhanced in a compacted lattice due to global ET rate enhancements.

Polystyrene quenching

In addition to cross relaxation, the green-emitting $^4S_{3/2}$+$^2H_{11/2}$ and red-emitting $^4F_{9/2}$ states can be depopulated via ET to high energy vibrational modes immediately outside the nanoparticle. These processes are operative across larger donor-acceptor separations because of the relatively large oscillator strengths of the allowed vibrational transitions of the surrounding matrix.[23] Rabouw et al. found that the $^4F_{9/2}$ decay rate was significantly reduced in predominantly aromatic (versus aliphatic) solvents, but the $^4S_{3/2}$ decay rate of nanoparticles with identical erbium concentrations was robust to solvent character. In a similar vein, Fischer et al. determined that the ratio of the surface quenching rate to the intrinsic depopulation rate (ie, in nanoparticles with sufficiently thick shells) is higher for the red emitting state (1.1) than it is for the green emitting state (0.8).[27] These results suggest that the solvent - or the chemical environment more generally - differentially quenches red emission at atmospheric pressure. In support of these findings, we show in ED Fig. 5 that the Raman-active aliphatic -CH stretch of polystyrene (~2912 cm^{-1}) has good overlap with the center of mass of the broad red quenching $^4F_{9/2}$→$^4I_{9/2}$ transition (labeled RQT, ~2850 cm^{-1}) tabulated for LaF_3 by Carnall et al.[24] and measured directly for flourindate glass via MWIR fluorescence spectroscopy.[28] The aromatic -CH stretch of polystyrene (~3060 cm^{-1}) has slightly worse energy matching with the center of mass of the broad, green quenching $^4S_{3/2}$→$^4F_{9/2}$ transition (labeled GQT, ~3150 cm^{-1}), which again is tabulated for LaF_3 by Carnall et al.[24] and has been measured for fluoroaluminate-tellurite glass via MWIR fluorescence spectroscopy.[29] Although our vibrational spectra were acquired via Raman spectroscopy (see Methods subsection “Diamond anvil cell preparation for Raman and UCL measurement”), these transitions are also IR active.[30]

Although polystyrene’s vibrational modes couple more strongly to the RQT at atmospheric pressure, they will broaden and blueshift at elevated pressure, improving resonance with the GQT and potentially enhancing I_{Red}:I_{Green}. From atmospheric pressure to 3.1 GPa , the polystyrene aromatic peak blueshifts by 25 cm^{-1}, towards the center of mass of the GQT (ED Fig. 5c-d). Pressure induced broadening

may enhance resonance further. This transition is also a prominent $^4F_{9/2}$ inflow, so its potential to enhance I_{Red}:I_{Green} at high pressures is twofold. Over this same range, the aliphatic peak(s) blueshift by 35 cm^{-1}, away from the center of mass of the RQT. The effect of this shift on RQT resonance is less clear because the RQT is likely to broaden at high pressures. Future pressure dependent MWIR fluorescence studies would clarify the trend in red emission quenching. It is worth noting that matrix quenching may have contributed to the I_{Red}:I_{Green} enhancements measured in previous DAC experiments despite the absence of polystyrene, because the silicone oil pressure medium exhibits a qualitatively similar trend in its resonance with the two quenching transitions (ED Fig. 5c-d). Non-radiative outflow from the green emitting state could be stimulated further by the simultaneous densification of these vibrational modes. Using the Tait equation for a polystyrene glass at 20°C (SI Eq. 4), we estimate that this density should increase by 4.5% over only 200 MPa (the pressure range for which the equation of state is strictly valid):

$$\rho_P = \frac{\rho_0}{1 - C \cdot ln(1 + \frac{P}{B(t=20)})} \qquad \text{SI Eq. 4}$$

where $C = 0.0894$, and $B(t = 20°C) = 3.267$ kilobar.[31,32] Overall, these data suggest that green emission may experience more efficient matrix quenching at high pressure.

Other mechanisms

There is also precedent in the literature to suggest that pressure mediates UCL response by distorting local symmetry, increasing the index of refraction (IOR) of the polystyrene matrix, and blueshifting lattice phonon modes. Wisser et al. show that hydrostatic pressures below ~2 GPa increase UCL intensity and decrease UCL lifetime in α-$NaYF_4$, but strictly decrease UCL intensity in β-$NaYF_4$, a distinction they explain in terms of local crystal field symmetry breaking.[33] We note that, although the actual point group for Ln^{3+} centers in the cubic lattice is likely not octahedral at the doping concentrations we use, it is still likely to be centrosymmetric (D_{4h}) at atmospheric pressure.[34] UCL radiative emission rates are also positively dependent on the IOR of the suspension medium.[23] For polystyrene, IOR will

scale linearly with hydrostatic pressure up to ~200 MPa, before dampening in response at higher pressures.[35,36] Finally, pressure will affect peak energies and widths for lattice phonon modes, but the impact this has on UCL is perhaps marginal. Runowski et al. measure a ~17 cm^{-1} blueshift in the phonon spectrum of cubic-phase SrF_2:Er,Yb over 5.29 GPa, but conclude it is too small of a shift to explain the red and green lifetime reduction for Er^{3+} UCL that they observe over that same pressure range.[37]

Section 7: Electrical and optical measurement of pharyngeal function

Signal synchronization: Trigger setup and lag

Two-channel (red, green) upconversion luminescence videos were acquired from ingested microgauges according to methods described in the main text in tandem with electrophysiological records of pharyngeal muscle activity (aka electropharyngeograms or EPGs). The data streams were synchronized using an Arduino Leonardo to trigger acquisition on both the camera (ORCA Flash4.0) and the EPG microfluidics chip. The camera was operated from a desktop PC (Dell Precision T3610, "camera computer") and the EPG and Arduino were operated from a laptop (Lenovo ThinkPad P1, "EPG computer"). The camera was operated in External Start Trigger mode, while the mouse cursor of the EPG computer was positioned to start the acquisition in NemAcquire, a software application provided by the EPG chip manufacturer (inVivo Biosystems). Once switched on, the Arduino delivered a TTL pulse via a BNC-to-SMA cable to initiate the camera acquisition and a left mouse click trigger (Mouse.click) via a USB cable. When operating the ORCA Flash4.0 in this triggering mode with an exposure time below 100 ms, the first exposure frame is dropped (Orca Flash4.0 Manual). This single dropped frame introduces a 20 ms lag between the receipt of the TTL trigger and the exposure of the first valid frame. There is a relatively negligible jitter of 30 μs. There is also a lag in the EPG computer's response to a mouse click

event; because of the nature of the task scheduler in the Windows 10 OS, this lag is non-deterministic. Below we discuss how this jitter results in a maximum two-frame uncertainty in the true offset between the recording streams.

Lag estimation

We used a simple method to measure the lag between the start of the camera and EPG acquisition (SI Fig. 13). To determine when a simultaneous electrical and optical signal would be registered on both recordings, we positioned an LED so that its emission would be captured by the camera and placed the wires driving the LED close enough to the chip's electrodes to generate an electrical signal. For each trial, we triggered a dual acquisition as above, and then manually switched the LED on and off at nearly regular intervals within the 60-second acquisition (50 Hz on the camera, 500 Hz on the EPG). In SI Fig. 13b, we plot the average pixel brightness and raw voltage time courses from one of the ten trials. In every trial, the relative time at which the camera registered the first optical signal preceded the relative time at which the EPG recording reflected the voltage change. Across 10 trials, we measured an average lag of 64 ms ±18 ms (min: 20 ms; max: 100 ms) (SI Fig. 13c). The temporal resolution (20 ms) of these measurements is limited by the camera frame rate (50 Hz). Thus, the average lag corresponds to three frames, with a one-frame uncertainty. We offset the optical time course by the average lag (three frames) when generating the event-triggered averages so that the maximum uncertainty in the coincidence with R would never be more than two frames in either direction. To confirm that a single lag offset could be applied to the entire time course without introducing systematic error within the trial, we also explored the correlation between the elapsed time and change in lag within individual trials (SI Fig. 13d). Any dropped frames (or voltage measurements) would result in an increase (or decrease) in the lag. We fit the change in lag relative to the first measured lag against the elapsed time within the trial according to a linear model with a fixed intercept of zero. Although there was a statistically significant correlation ($p = 5 * 10^{-5}$) between the two, the slope

was only 0.17 ms/s (SE = 0.04 ms/s). This means that over the course of the 60 s, 3000 frame recording, lag between the optical and electrical signals is expected to increase by only 10.2 ms. The effect of this expected error on our measurements is negligible since it is roughly half of the acquisition time for a single imaging frame.

Event-triggered averages

Optical data processing

We converted raw UCL videos into time series of the total red and green emission from the terminal bulb of ingested microgauges in worms held in the EPG chip as follows. The general strategy was to locate microgauges in the red channel (which is the brighter of the two UCL channels) and to analyze the total intensity in both channels in this region of each frame. To reach this goal, in each frame, the red channel was binarized using a threshold pixel intensity calculated by the Sobel method and manually scaled to account for the variations in microgauge accumulation in the anterior intestines of individual worms. This binarized image was dilated with an octagonal kernel and eroded with a diamond kernel and kernel sizes were adjusted manually in order to achieve the best possible separation among regions of interest (ROIs) in the terminal bulb (imaging target), anterior intestine, and anterior pharynx. Images were filtered and bridged (to recover missed targets) using a 3D cuboidal kernel of size [x = 1, y = 1, frame = 5]. Of the three ROIs, the intestine was generally the largest and this size differential allowed the analysis to focus on the ROI associated with the terminal bulb. In a minority of frames, the intestinal ROI obscured the terminal bulb, leaving a gap in the time-series of UCL emission from the pharyngeal bulb. These were bridged, where possible, using the aforementioned 3D kernel. We measured the UCL intensity in the red and green channels for the terminal bulb ROI, generating a time-series of I_{Red}:I_{Green} emission from microgauges transiting through the terminal bulb of the pharynx. We adjusted the resulting time series for the temporal lag between camera and EPG acquisition by culling three frames from the

beginning of the optical time series, and a corresponding 60 ms worth of data from the end of the electrical time series.

EPG data processing

To average electrical signals associated with a single pharyngeal pumping cycle, EPG traces were divided into individual pump events as follows. First, we identified a time window that would include the excitation or E phase, the relaxation or R phase, the interval in between these signals and an equal amount of time before the E phase and following the R phase. Based upon the average pump duration (104 ms rounded to 100 ms) and the average pumping frequency (0.88 Hz), the resulting time window was 460 ms in duration (SI Fig. 15). We refer to the span of time from 20 to 180 ms as the post-R epoch of the event window. Analogously, the span from -280 to -120 is labeled as the pre-E epoch. A 20 ms buffer was included to allow for the voltage of each transient to reach baseline. Next, voltages measured in the two epochs were averaged and used to scale the entire event window. For worms that were recorded inverted in the channel (ie, with their pharynx facing the back electrode), we then applied a scaling factor of -1 to the zeroed voltage trace. This baseline shift and sign correction were the only data processing steps performed on the electrical time series.

The quality of the voltage trace was used as a proxy for event quality that could be calculated independently of the optical time series. Not only did this help us identify and remove falsely flagged R events, but it also reflects how stably the worm pharynx was seated in the channel. Low-frequency signals with magnitudes comparable to that of R could indicate motion parallel to the immobilization channel or perpendicular to it, as well as background electrical interference. As a measure of event quality, we calculate the root mean square error of the baseline corrected voltage in two windows at either end of the event window. We call these 80 ms periods the RMS windows (SI Fig. 15). The positioning of the RMS window in the post-R epoch (100 to 180 ms) was chosen to allow for any R2 transients to reach a baseline

voltage level. The RMS window in the pre-E epoch (-280 to 200 ms) was chosen so that it was equal in width to the other RMS window and could account for pump durations that were longer than the average. In other words, choosing to buffer the RMS window such a distance from the signal epoch prevents pump-inducing voltages from being counted in the RMS error in all recorded events. An RMS value greater than 20% of the magnitude of the R peak resulted in event exclusion (see Exclusion Criteria).

As a final note, we chose this simpler strategy to estimate event quality because it required fewer assumptions. Attempts to isolate the RMS of the "noise" from the pump-inducing signals via high pass filtering required us to make arbitrary assumptions as to frequency of the noise and resulted in values that still correlated with the magnitude of the signal in the pre-E and post-R epochs. Strategies based on polynomial fitting or moving averages suffered similar limitations. The relative magnitude of R provides an independent metric of signal that should be much larger than the magnitude of any feature in the pre-E or post-R epochs in which there is no pharyngeal muscle activation.

Event-triggered averaging of optical and electrical signals

To generate the event-triggered averages, we isolated individual pumping events from the EPG and the corresponding recording of I_{Red}:I_{Green} in the terminal bulb of the pharynx, which is proportional to the force applied to microgauges in this location. The peak of the R phase was set as $t = 0$ and the time axis was normalized to the interval in between the E and R phases. The force recording was normalized to the I_{Red}:I_{Green} value or "ambient force" observed during the E phase ($t = -1$), yielding $\Delta\%I_{Red}$:I_{Green} across the event. Using an emission ratio links these measurements to force via our AFM-confocal calibration and circumvents the need to account for differences in collection efficiencies between widefield optics used to measure microgauge activity *in vivo* and the confocal optics used to calibrate mechanosensitivity, if present.

Exclusion criteria

Several factors affect the quality of the imaging and EPG data streams obtained from individual animals loaded into the EPG chip. These include variations in microgauge ingestion, the need to rapidly load each worm into the EPG chip, and the quality of the immobilization. In total, we collected 92 one-minute, 3000-frame videos from 74 individual worms. However, many videos seemed to lack sufficient microgauge accumulation in the pharynx, as evidenced by low green emission. This situation likely reflects that ingested microgauges are continually transported from the pharynx to the intestines from the moment animals are removed from the feeding plate and loaded into the EPG chip. Delays in this process and in laser alignment result in few, if any microgauges being present in the pharynx when video acquisition is started (SI Fig. 16a). Additionally, some worms would slip within the immobilization channel, independent of the stability of the initial immobilization (SI Fig. 16d). Occasional anterior body contractions or lateral motions of the pharynx resulted in sudden, large movements of the terminal bulb that impaired image segmentation. Finally, there were several instances where the noise in the EPG signal exceeded the ability of the manufacturer's NemAnalysis software to locate the E/R transients. Forty-nine (49) videos that showed evidence of low signal, instability of the worm or pharynx in the EPG chip, or excessive noise in the EPG signal were excluded before any segmentation was attempted.

Of the remaining 43 videos, 22 were segmented using our image processing pipeline. Videos could be segmented if image analysis parameters could be chosen to capture the terminal bulb and exclude the anterior pharynx and intestines in all frames (SI Video 6). In the 21 videos that could not be segmented, the terminal bulb was too close to the intestinal signal (SI Fig. 16b), the isthmus had a significant signal for a large portion of the video, or the terminal bulb signal was too low compared to that of the intestines and the anterior pharynx. Two hours of parameter optimization were attempted on each video before

attempts at segmentation were abandoned. This decision was independent of any knowledge as to the magnitude or direction of I_{Red}:I_{Green} change in the terminal bulb, since segmentation is required to obtain that information.

The quality of the segmentation was rated by three volunteers. In brief, outlines of the segmented pixels were overlaid on each video and coded videos were viewed by volunteers who rated the videos on a scale of 1 to 5 based on three criteria: 1) the frequency and magnitude with which terminal bulb signal breached the segmentation outline during a pump (SI Fig. 16c), 2) the average proportion of terminal bulb signal that was excluded from the outline across all frames, and 3) the proportion of the total segmented pixel region that comprised either the intestines, isthmus or anterior pharynx. A score of one (1) represented a segmentation with frequent breaches that did not fully capture the terminal bulb and which often captured signal outside of the terminal bulb, while a score of five (5) represented a segmentation that fully and exclusively captured the terminal bulb signal. Individual scores were averaged, and all videos receiving a score of two (2) or less were excluded. These threshold criteria were not provided to scorers. Seven (7) videos were excluded based on this criteria, leaving fifteen (15) videos with high-quality segmentation. Imaging pipelines that make use of machine-learning based tools for image segmentation are emerging as a more robust tool than intensity-based segmentation and could harvest more information from this data set in the future and from additional similar data.

Although the selection of a 460 ms event window was appropriate given the pump durations and pumping frequencies exhibited by the vast majority of the worms, it posed an issue for individual worms pumping faster than 2 Hz on average. These individuals would exhibit R (and E) transients in nearly all of the pre-E (and post-R) epochs. These events would then be flagged for exclusion based on our previously determined criteria. Thus, we excluded four data sets that had an average pumping frequency above 2 Hz rather than including the negligibly few events where there was no signal interference from

the previous or subsequent event. A second frequency exclusion criteria put the minimum acceptable pumping rate at 0.167 Hz, or 10 pumps in 60 seconds. It was assumed that any worm pumping slower than this in the presence of serotonin was not representative. Four (4) additional data sets were excluded based on this criteria, leaving the final remaining total of seven data sets. Each came from a separate worm, and spanned three of the five days of study. From the seven worms, a total of 61 events were excluded based on the RMS criteria, leaving a final event total of 185. As an example, all 12 of the 30 total pumping events corresponding to Fig. 4c.iv and SI Video 6 that did not pass RMS quality control are displayed in SI Fig. 16e.

Section 8: Supplementary Figures

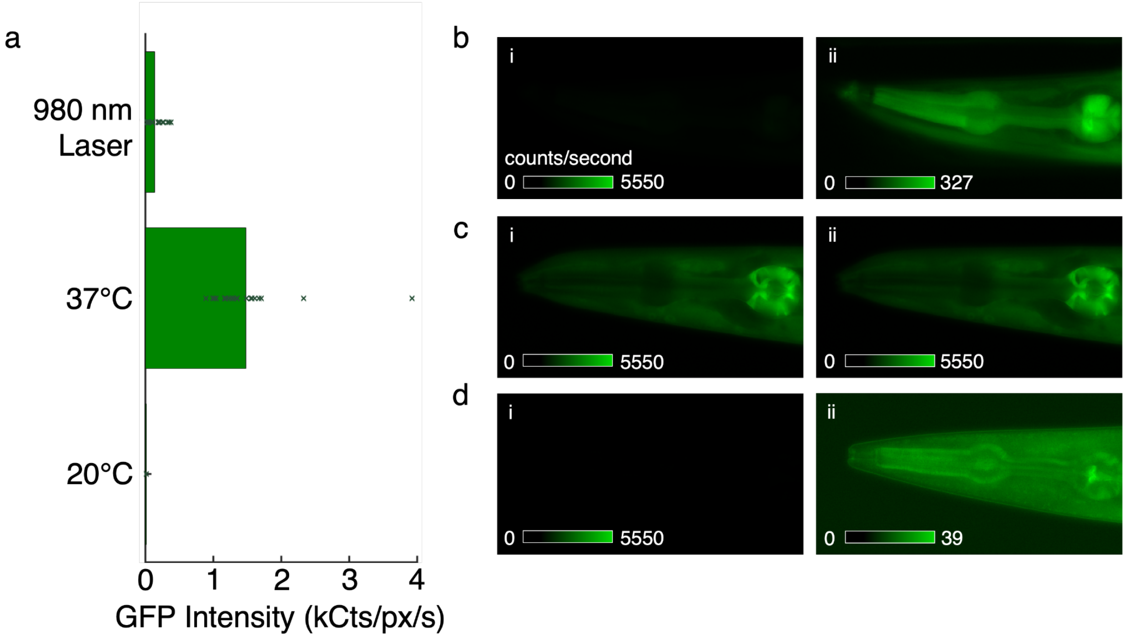

SI Fig. 1: 980 nm laser irradiation induces modest thermal stress

a) Average GFP intensities in the heads of TJ375 *hsp-16.2::GFP* transgenic worms 16 hours after a one-minute exposure to a 13 kW/cm^2 980 nm laser (top, 143 Cts/px/s), a 2 hour incubation at 37°C (middle, 1483 Cts/px/s), a 2 hour incubation at 20°C (bottom, 17 Cts/px/s) and N=21 for all three conditions. **b-d**) Representative fluorescence images of GFP expression in the three conditions at the same scale (980 nm laser: **bi**, 37°C: **ci**, 20°C: **di**) and on separate scales (980 nm laser: **bii**, 37°C: **cii**, 20°C: **dii**). In the control condition, there is a significant luminal component from ingested bacteria. The contribution is small in the other two conditions, and the lumen appears relatively dark.

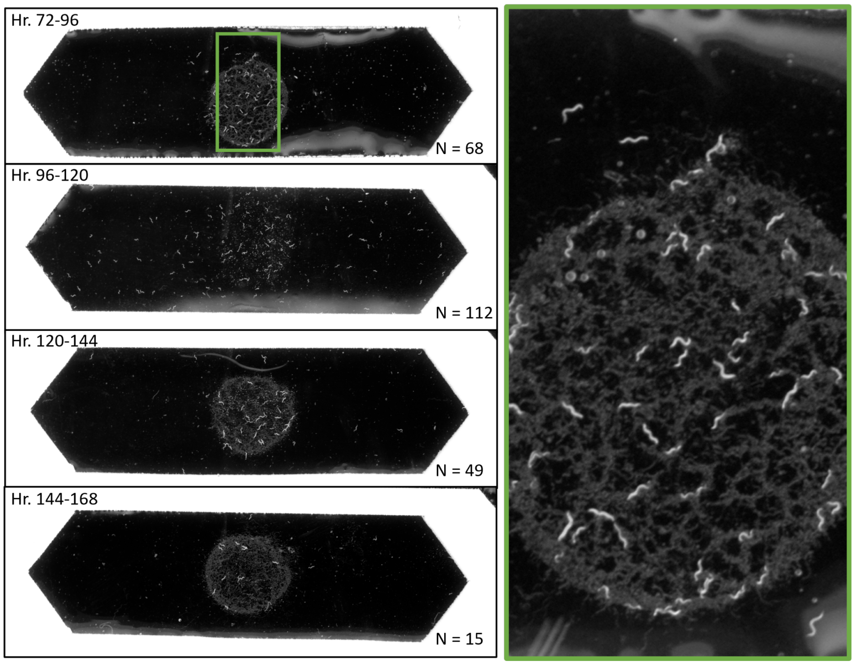

SI Fig. 2: Egg-laying wells

Scanned images of egg-laying wells that were used to count the number of progeny produced within each 24-hour period. All four images correspond to the same egg-laying individual, which belonged to the microgauge-fed test group. The hours since bleaching and the number of worms counted are labeled in the upper-left and lower-right corners of each image, respectively. The white boundary is a cut foam insert that discourages worms from crawling out of the arena.

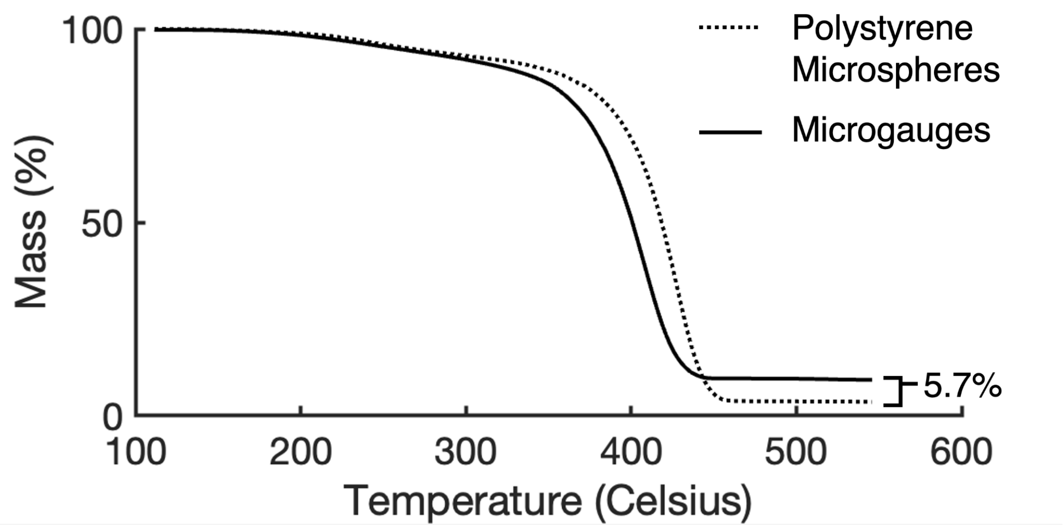

SI Fig. 3: Thermogravimetric Analysis of UCNP mass fraction in microgauges

A plot of residual mass as a function of temperature for polystyrene embedded $NaY_{0.8}Yb_{0.18}Er_{0.02}F_4$@$NaYF_4$ (microgauge, solid line, initial mass 5.04 mg) and polystyrene microspheres only (dashed line, initial mass 19.007 mg). Both mass percentages are referenced to the mass remaining after a 20-minute hold at 110℃ to account for residual moisture.

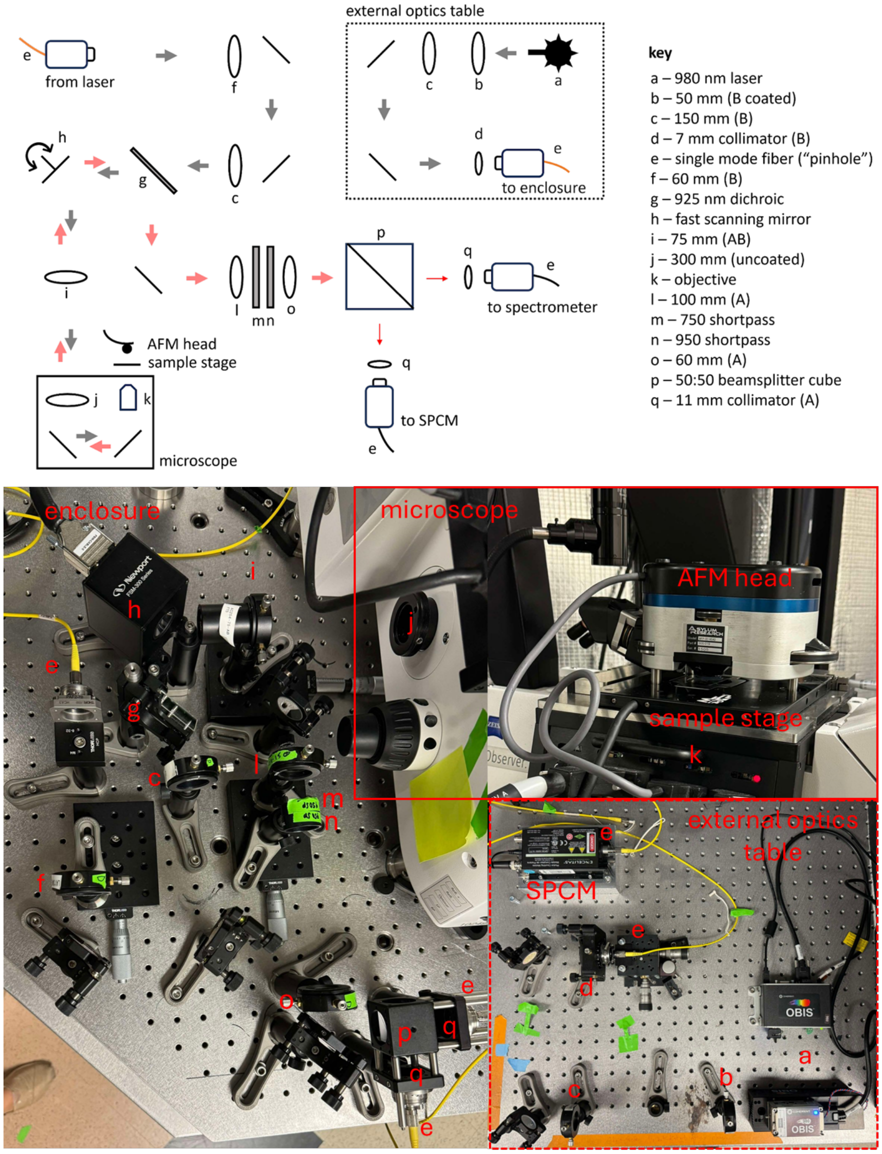

SI Fig. 4: Confocal optical train

A schematic diagram (above) and photographs (below) of the confocal optical train. Gray arrows represent the 980 nm excitation path, and red arrows represent the visible (red and green) collection path. All components are housed in a sealed vibration isolation chamber except for those represented inside the dotted outline. Single-mode collection fibers perform the function of the confocal pinhole.

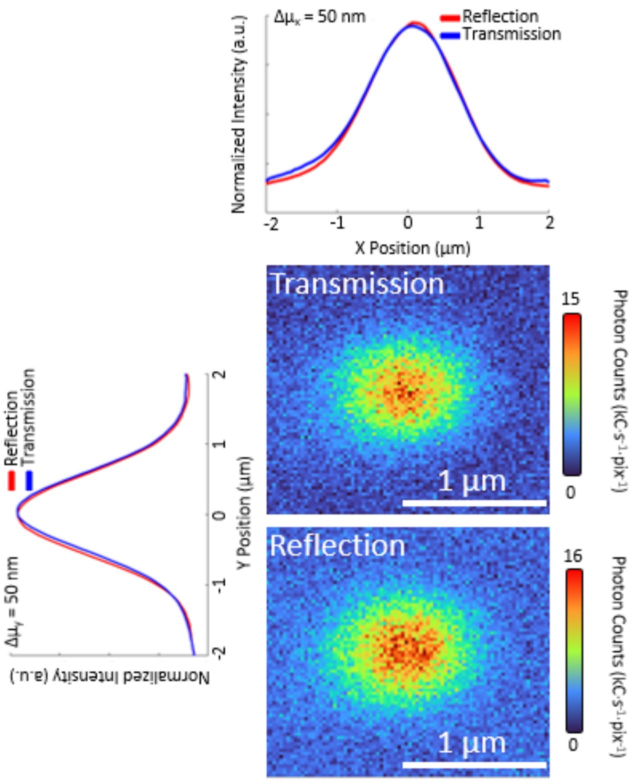

SI Fig. 5: Co-alignment of two confocal channels

UCL maps of the same single UCNP taken with the same field of view and focal height showing the co-alignment of two imaging channels downstream of a 50:50 beamsplitter. Images were taken with the same fiber-coupled SPCD, switching between the transmission and reflection fibers. Row and column averages from ten images per channel were fitted to a Gaussian to estimate peak offset in each direction. One pixel offset in both X and Y, representing 50 nm in both directions and ~70 nm total, was measured.

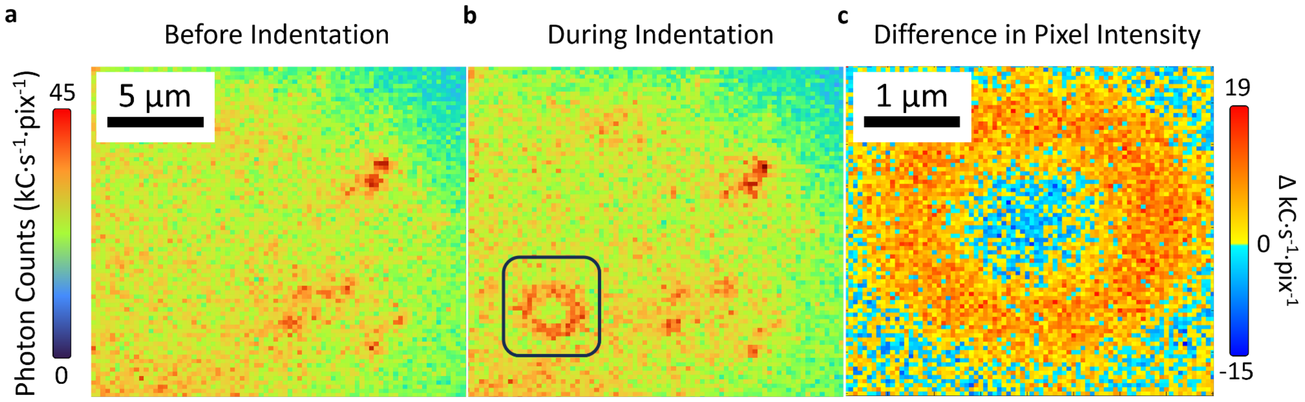

SI Fig. 6: Colocating the centers of the confocal excitation and the contact region

a) A UCL map taken on the reflection channel of the confocal microscope before indenting the sample with a 10 μm colloidal tip at a trigger force of 1 μN. **b**) During the indentation. Colormaps, scale bars, and fields of view are identical for a and b. **c**) The difference in pixel intensity from the contact region after indentation relative to pixel intensities before indentation.

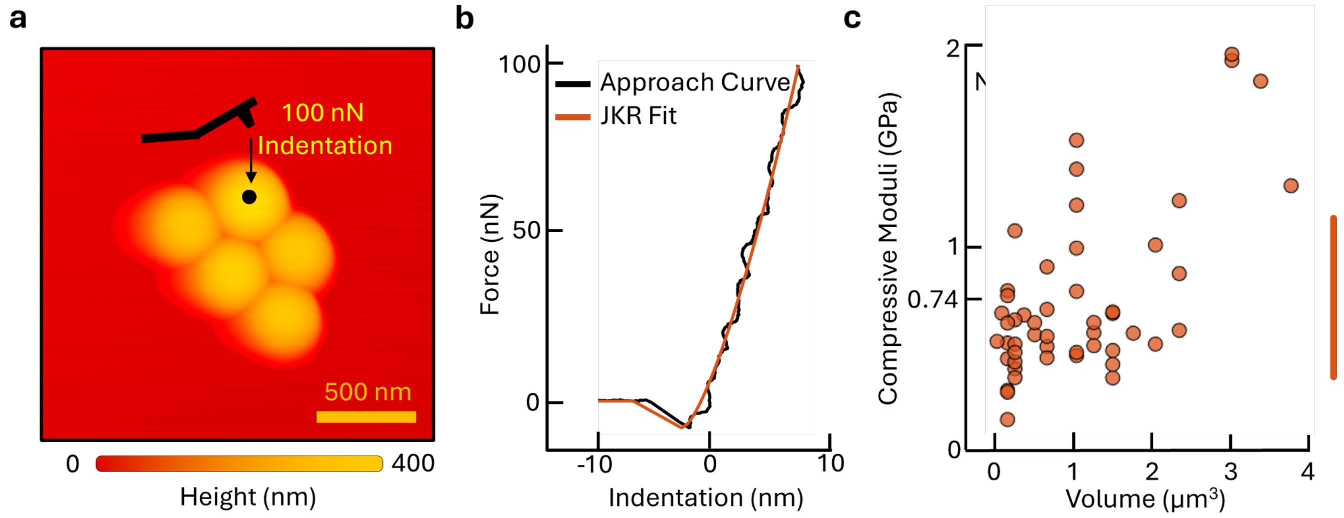

SI Fig. 7: Method for estimating microgauge compressive modulus

a) An AFM height map taken with a 30 nm radius rounded tip operating in AC mode, showing a cluster of individual microgauges. **b**) A force indentation curve generated from a single microgauge indented to a trigger force of 100 nN (black) and fitted to a JKR model (orange). **c**) All compressive moduli obtained by fitting the approach curves of 50 indented microgauges displayed as a function of particle volume, with the mean (740 MPa) and standard deviation (410 MPa) indicated to the right.

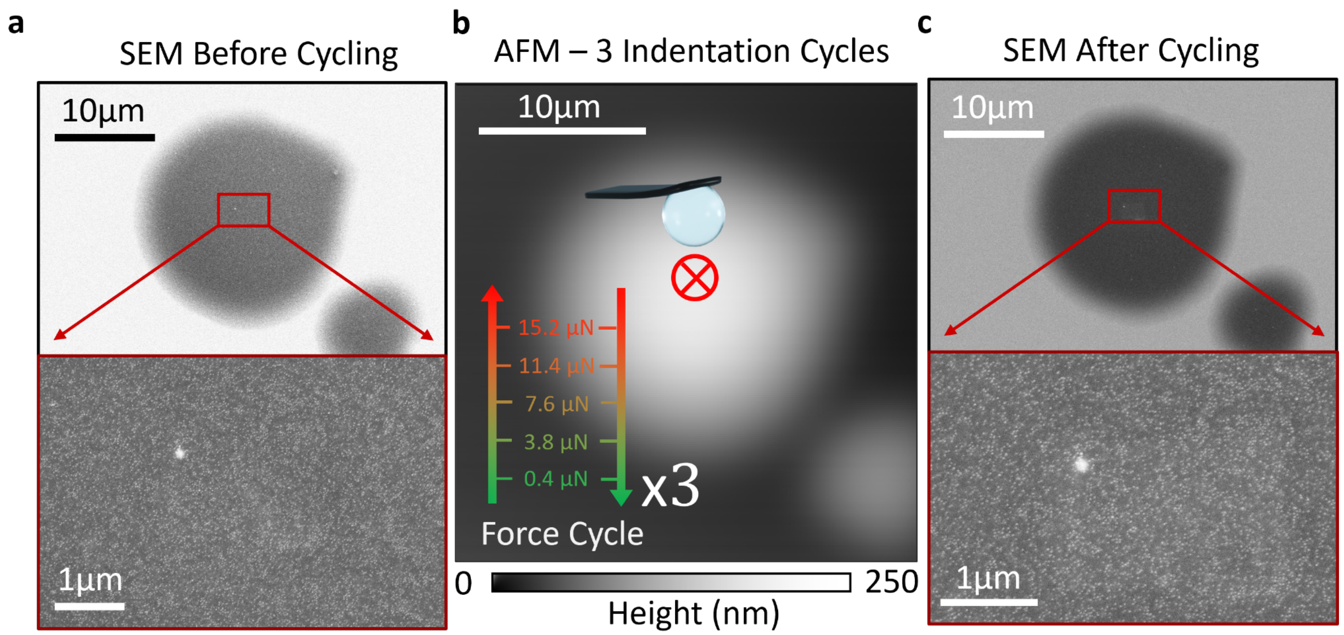

SI Fig. 8: Microgauge films do not exhibit plastic deformation under the indentation conditions used for optical calibration

a) SEM micrograph of an uncoated patch of microgauge film on silicon taken before indentation, and a higher resolution inset at the indentation region. **b**) A height map of the same patch taken with the 10 µm diameter colloidal tip operating in AC mode. The region indicated with a red X was indented with the same tip operating in contact mode from 0.4 to 15.2 µN and back down again. Each force was held for 30 seconds, and three loading-unloading cycles were performed before withdrawing the tip. **c**) SEM micrograph of the same patch taken after the indentation cycles and a higher resolution inset at the indentation region. The faint rectangular patch of contrast is from beam-induced deposition of organics within the SEM, which occurred during pre-indentation imaging.

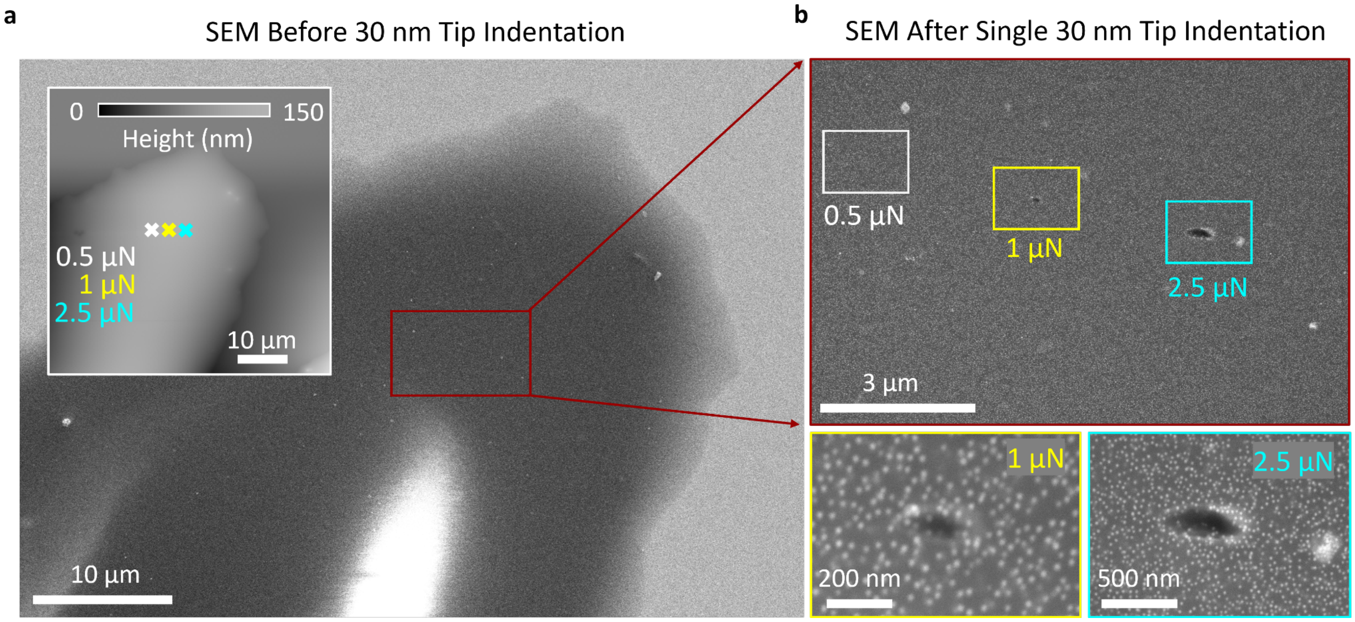

SI Fig. 9: Microgauge films do exhibit plastic deformation at indentations in excess of the compressive strength of polystyrene.

a) SEM micrograph of a large uncoated patch of microgauge film on silicon before indentation. The inset shows the AFM height map of the same region taken with a 30 nm radius rounded tip operating in AC mode as well as the locations and magnitudes of indentation forces. **b**) SEM micrographs of the same region after indentation, with higher resolution insets showing the film damage occurring at 1 and 2.5 µN (~510 and 700 MPa maximum contact stress, respectively). No scarring was observed in the region around the 0.5 µN indentation, corresponding to ~400 MPa maximum contact stress.

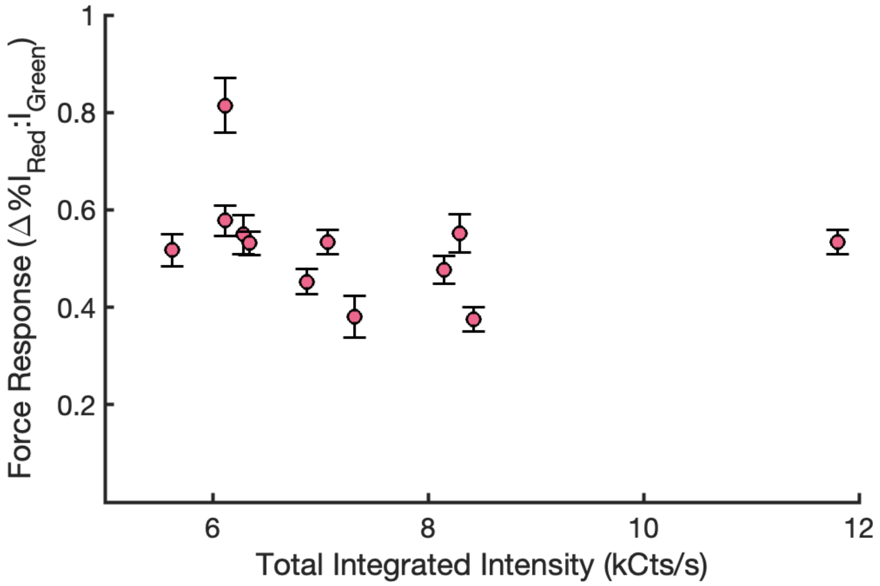

SI Fig. 10: Correlation between force response and UCNP loading

The slope of the least squares fit of percent ratiometric change versus force for all 3 cycles of each replicate plotted against the average total integrated intensity in the red and green regions (I_{Red}+I_{Green}) for three 90 second integrations taken with the tip engaged at 0.4 μN prior to the indentation cycles. Error bars represent the S.E.

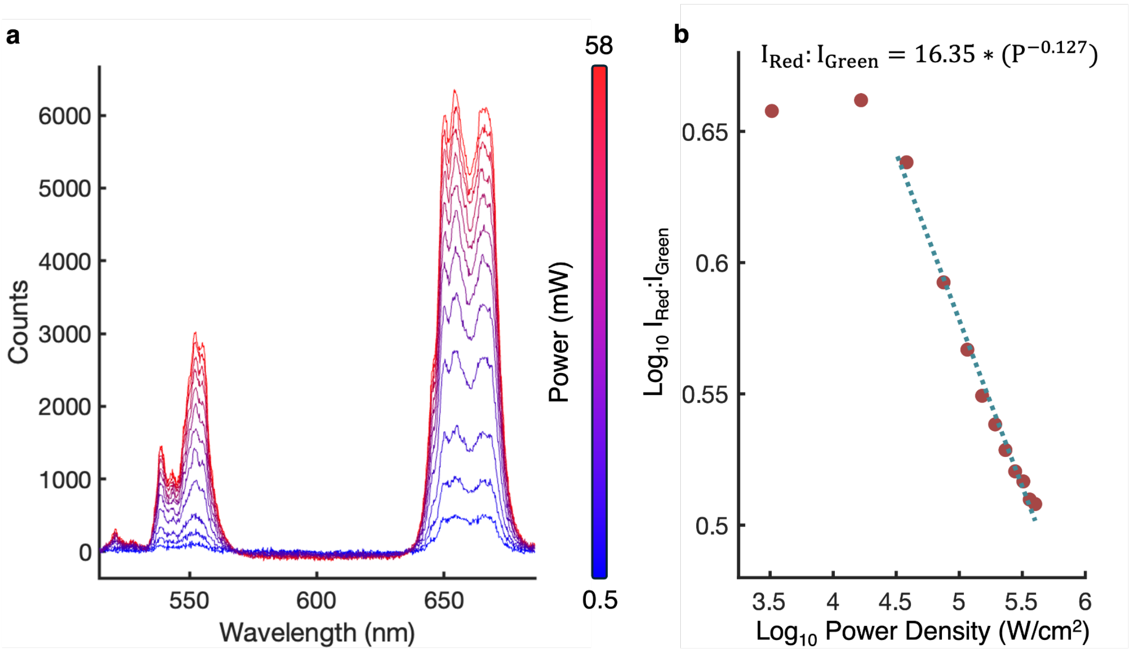

SI Fig. 11: I_{Red}:I_{Green} *vs.* incident laser power

a) Background corrected confocal UCL spectra from a single microgauge taken at different incident powers (0.5 to 58 mW, exiting objective). **b**) A log-log plot of I_{Red}:I_{Green} values extracted from the spectra in panel a, fit to a broken power law above 32 kW/cm^2.

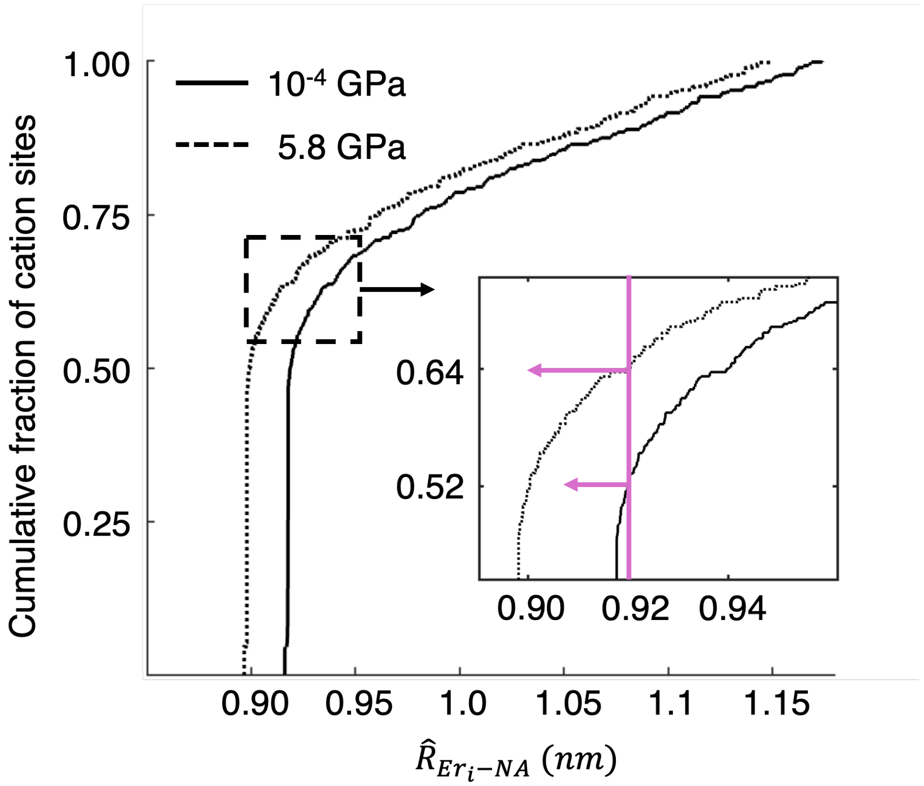

SI Fig. 12: Expected nearest erbium acceptor distances

The cumulative distribution function of expected nearest erbium acceptor distances for all cation sites in a 10.9 nm diameter $NaY_{0.8}Yb_{0.18}Er_{0.02}F_4$ core. The calculation was performed at atmospheric pressure (solid) and the maximum pressure employed in the UCL DAC study (5.8 GPa, dashed). The vertical magenta line in the inset indicates the critical Förster radius for cross relaxation determined by [**Rabouw et al. 2018**], and the horizontal magenta arrows are the fraction of sites at each pressure expected to have nearest neighbors closer than that threshold.

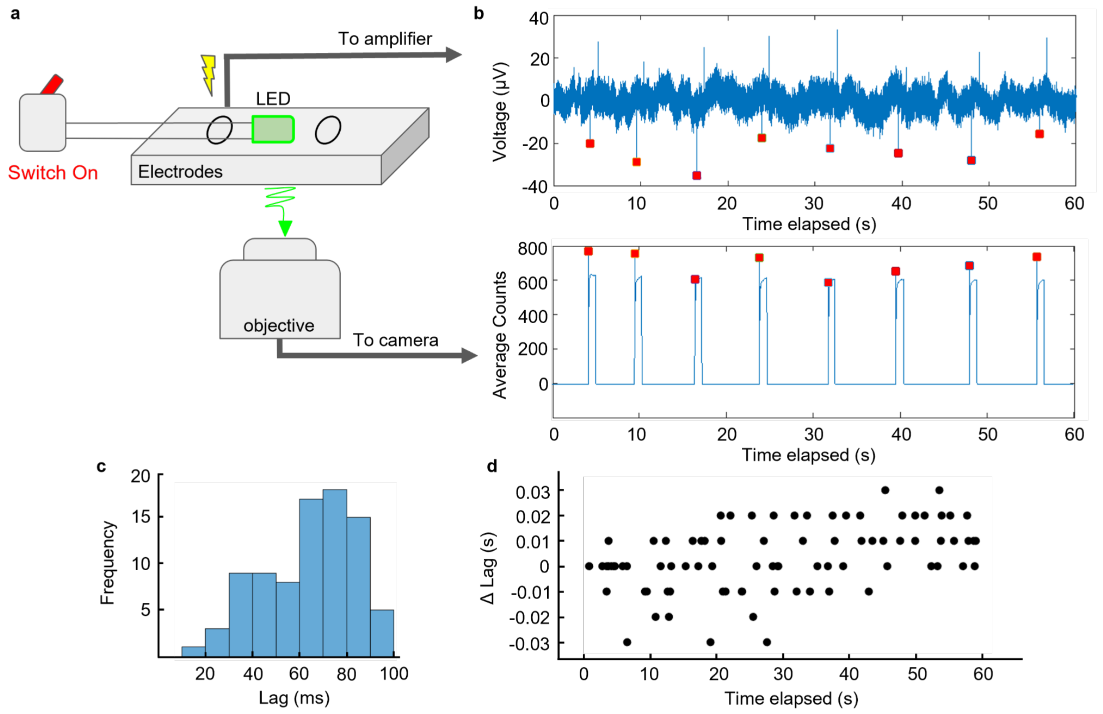

SI Fig. 13: Lag estimation

a) A schematic diagram of the lag measurement setup wherein a simultaneous electrical and optical impulse are delivered to the two recording streams. The switch was toggled manually between seven and ten times per 60-second trial. **b)** The relative time course of voltage measured on the EPG chip and average pixel brightness measured on the camera from a sample trial. Red squares are used to denote the point on each time course when a change was initially observed. **c)** A histogram of all lags (of the EPG relative to the camera) measured across all ten trials. Each bin has a 10 ms width, and the label represents the upper limit for the bin (inclusive). The minimum lag we observed was 20 ms, and the maximum was 100 ms. **d)** The same lag measurements plotted as their relative change from the initial observed lag within their respective trial. This data represents how synchronicity is affected by data loss in one or both of the recording streams. We note that the units on the x and y axes are both seconds, but the scale of the y axis is drastically exaggerated for clarity.

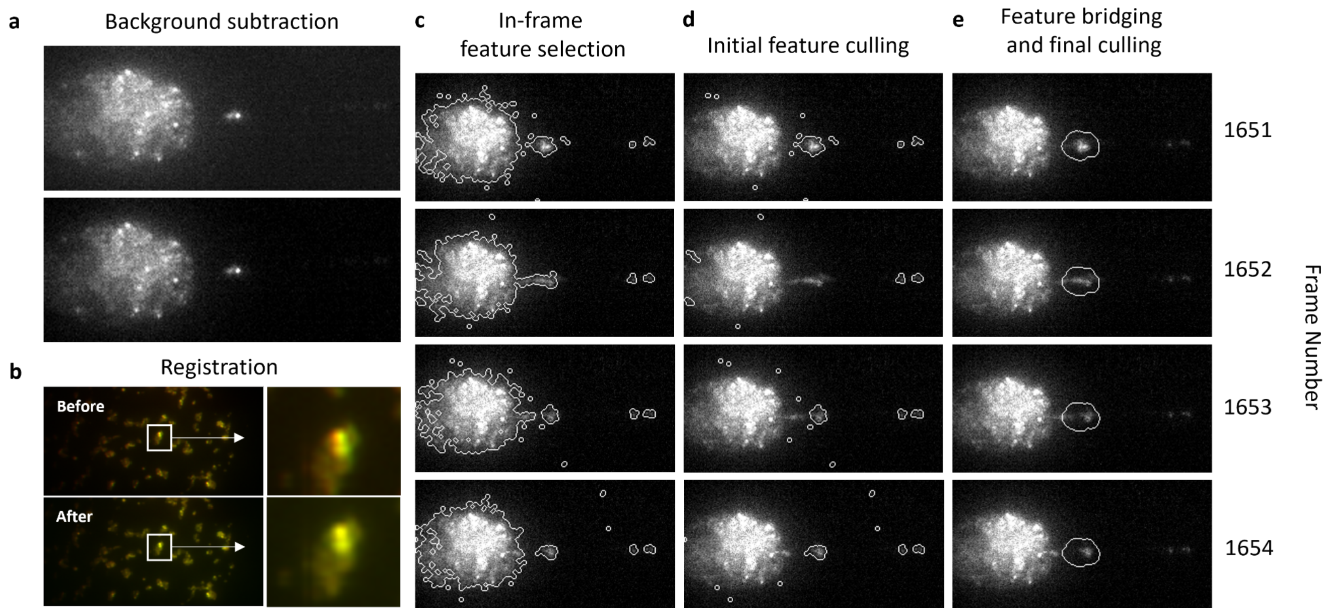

SI Fig. 14: Optical data processing pipeline

a) One example frame from the red channel of a recorded worm (Fig. 4c.iv) before and after subtracting the background image **b)** An overlay of the red and green channel of the reference image taken after the W-View Gemini lens alignment and before the recording (before), as well as the same image overlay after translational registration (after). **c)** Example frames 1651-1654 from the same recording after the initial binarization and 2D morphological image processing. Outlines represent all contiguous features that survived the thresholding and opening. Notice that in frame 1652, the terminal bulb feature is contiguous with the intestinal feature because material is passing through the VPI into the intestines under the influence of a pump. **d)** The same frames after removing the feature with the largest area, which almost always contains the intestinal signal. Notice that this also erroneously culls the terminal bulb signal in frame 1652. **e)** The same frames after 3D morphological image processing to bridge lost terminal bulb features (ex. 1652) and remove noise. The outline

depicted in frame 1652 was thus interpreted from the spatial information of the features in a set of neighboring frames. If, as in this case, the anterior pharynx features survived this process, they were removed manually. The segmentation output for this 3000 frame recording is presented in full in SI Video 6.

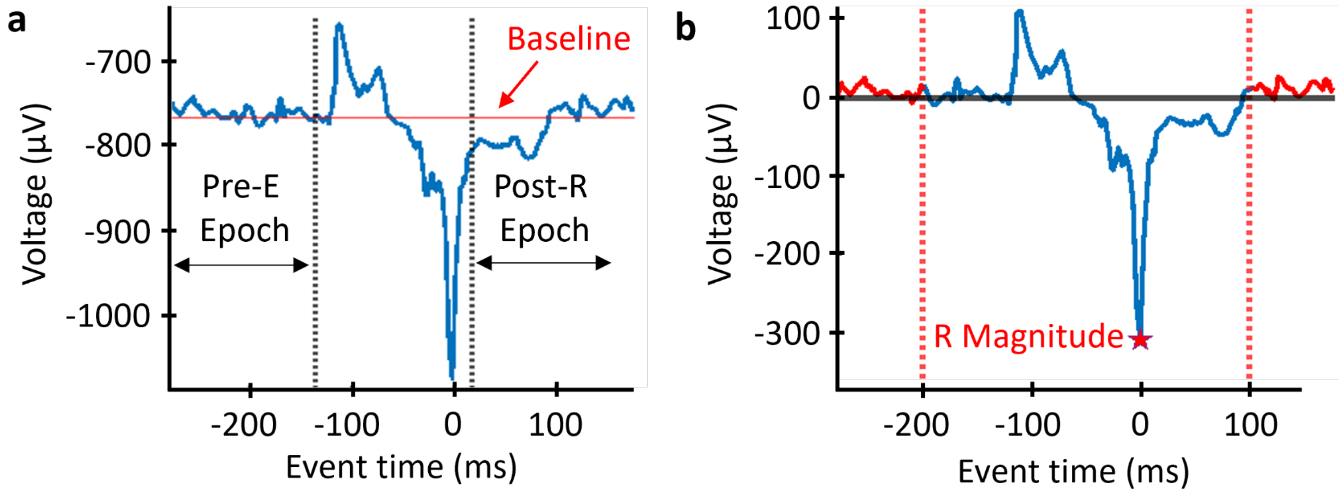

SI Fig. 15: Electrical baseline subtraction

An illustration of the event window before time normalization as well as the voltage time course from a sample pump **a**) before and **b**) after DC offset. This baseline correction is calculated from the mean of the Pre-E and Post-R Epochs. Once corrected, the RMS of the event noise (red regions) is calculated and compared to the magnitude of the R peak. As illustrated, in the absence of low-frequency interference, this value should be well below the 20% threshold used for quality control. See SI Fig. 16e for examples of the voltage-time courses that do not pass quality control.

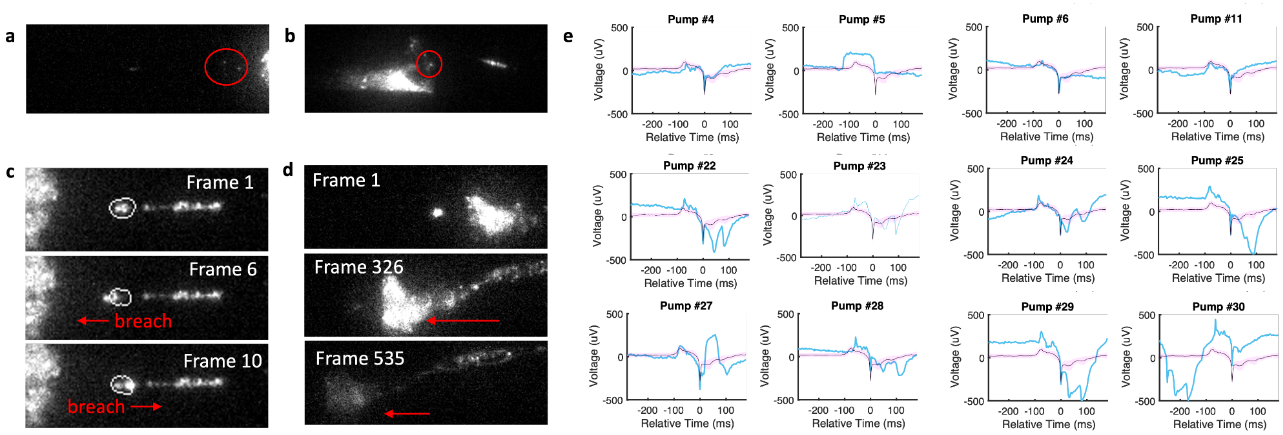

SI Fig. 16: Exclusion criteria

An illustration of several conditions that resulted in optical data set exclusion either **a,b**,**d** before or **c**, after segmentation, as well as the individual events that were excluded based on the electrical data set within a sample data set (Fig. 4c.iv) that did pass quality control. **a)** A frame illustrating a lack of sufficient terminal bulb accumulation (red circle), as well as a lack of accumulation in the anterior pharynx to eventually replenish it. **b)** A frame illustrating the difficulty of isolating terminal bulb signal (red circle) in the morphological image processing step when the terminal bulb is persistently too close to the intestines. **c**) A representative set of three frames from a segmentable data set that was given an average, blinded segmentation score of 1.3. As illustrated, this data set exhibited frequent segment “breaches" during pumps, which resulted in the measured ratiometric changes not being representative of those in the terminal bulb as a whole. Such breaches usually occur in recordings where the segment needs to be made small enough to avoid capturing luminescence from microgauges accumulated in the anterior isthmus. **d)** Three frames illustrating the progressive backward drift of a worm. Aside from insufficient accumulation, this was the most commonly observed condition that resulted in data set exclusion. **e)** All 12 events (blue) in the order they were observed that were excluded from Fig 4c.iv because of an RMS value that exceeded 20% of the R

magnitude. “Relative time” is used synonymously with “event time.” The average (black) and standard deviation (magenta) of the 18 surviving events are overlain for comparison.

Supplementary Citations

1. Rea, S. L., Wu, D., Cypser, J. R., Vaupel, J. W. & Johnson, T. E. A stress-sensitive reporter predicts longevity in isogenic populations of Caenorhabditis elegans. *Nat. Genet.* **37**, 894–898 (2005).
2. Guo, D., Li, J., Xie, G., Wang, Y. & Luo, J. Elastic properties of polystyrene nanospheres evaluated with atomic force microscopy: size effect and error analysis. *Langmuir* **30**, 7206–7212 (2014).
3. Tan, S., Sherman, R. L., Jr & Ford, W. T. Nanoscale compression of polymer microspheres by atomic force microscopy. *Langmuir* **20**, 7015–7020 (2004).
4. Rutland, M. W., Tyrrell, J. W. G. & Attar. Analysis of atomic force microscopy data for deformable materials. *J. Adhes. Sci. Technol.* **18**, 1199–1215 (2004).
5. Johnson, K. L., Kendall, K. & Roberts, A. D. Surface energy and the contact of elastic solids. *Proc. R. Soc. Lond.* **324**, 301–313 (1971).
6. Guruprasad, T. S., Bhattacharya, S. & Basu, S. Size effect in microcompression of polystyrene micropillars. *Polymer* **98**, 118–128 (2016).
7. Johnson, K. L. *Contact Mechanics*. (Cambridge University Press, 1987).
8. Politi, Y. *et al.* A spider’s fang: How to design an injection needle using chitin-based composite material. *Adv. Funct. Mater.* **22**, 2519–2528 (2012).
9. Duminis, T. *et al.* On the Mechanical Properties of Microfibre-Based 3D Chitinous Scaffolds from Selected Verongiida Sponges. *Mar. Drugs* **21**, (2023).
10. Sun, J., Wu, W., Ling, M., Bhushan, B. & Tong, J. A dynamic nanoindentation technique to

investigate the nanomechanical properties of a colored beetle. *RSC Adv.* **6**, 79106–79113 (2016).

11. Siamantouras, E. *et al.* Quantification of bush-cricket acoustic trachea mechanics using Atomic Force Microscopy nanoindentation. *Acta Biomater.* **153**, 399–410 (2022).
12. Sparacio, A. P., Trojanowski, N. F., Snetselaar, K., Nelson, M. D. & Raizen, D. M. Teething during sleep: Ultrastructural analysis of pharyngeal muscle and cuticular grinder during the molt in Caenorhabditis elegans. *PLoS One* **15**, (2020).
13. Donsì, F., Ferrari, G., Lenza, E. & Maresca, P. Main factors regulating microbial inactivation by high-pressure homogenization: Operating parameters and scale of operation. *Chem. Eng. Sci.* **64**, 520–532 (2009).
14. Wuytack, E. Y., Diels, A. M. J. & Michiels, C. W. Bacterial inactivation by high-pressure homogenisation and high hydrostatic pressure. *Int. J. Food Microbiol.* **77**, 205–212 (2002).
15. Auer, G. K. & Weibel, D. B. Bacterial Cell Mechanics. *Biochemistry* **56**, 3710–3724 (2017).
16. Vega, N. M. & Gore, J. Stochastic assembly produces heterogeneous communities in the Caenorhabditis elegans intestine. *PLoS Biol.* **15**, e2000633 (2017).
17. Lay, A. *et al.* Bright, Mechanosensitive Upconversion with Cubic-Phase Heteroepitaxial Core–Shell Nanoparticles. *Nano Lett.* **18**, 4454–4459 (2018).
18. Lay, A. *et al.* Upconverting Nanoparticles as Optical Sensors of Nano- to Micro-Newton Forces. *Nano Lett.* **17**, 4172–4177 (2017).
19. McLellan, C. A. *et al.* Engineering Bright and Mechanosensitive Alkaline-Earth Rare-Earth Upconverting Nanoparticles. *J. Phys. Chem. Lett.* **13**, 1547–1553 (2022).
20. Teitelboim, A. *et al.* Energy Transfer Networks within Upconverting Nanoparticles Are Complex Systems with Collective, Robust, and History-Dependent Dynamics. *J. Phys. Chem. C* **123**, 2678–2689 (2019).

21. Chan, E. M., Gargas, D. J., Schuck, P. J. & Milliron, D. J. Concentrating and recycling energy in lanthanide codopants for efficient and spectrally pure emission: the case of NaYF4:Er3+/Tm3+ upconverting nanocrystals. *J. Phys. Chem. B* **116**, 10561–10570 (2012).

22. Johnson, N. J. J. *et al.* Direct Evidence for Coupled Surface and Concentration Quenching Dynamics in Lanthanide-Doped Nanocrystals. *J. Am. Chem. Soc.* **139**, 3275–3282 (2017).

23. Rabouw, F. T. *et al.* Quenching Pathways in NaYF4:Er3+,Yb3+ Upconversion Nanocrystals. *ACS Nano* **12**, 4812–4823 (2018).

24. Carnall, W. T., Crosswhite, H. & Crosswhite, H. M. *Energy Level Structure and Transition Probabilities in the Spectra of the Trivalent Lanthanides in LaF_3*. http://dx.doi.org/10.2172/6417825 (1978) doi:10.2172/6417825.

25. Zhang, J. *et al.* Observation of efficient population of the red-emitting state from the green state by non-multiphonon relaxation in the Er3+–Yb3+ system. *Light Sci. Appl.* **4**, e239–e239 (2015).

26. Dong, H. *et al.* Efficient tailoring of upconversion selectivity by engineering local structure of lanthanides in Na(x)REF(3+x) nanocrystals. *J. Am. Chem. Soc.* **137**, 6569–6576 (2015).

27. Fischer, S., Bronstein, N. D., Swabeck, J. K., Chan, E. M. & Alivisatos, A. P. Precise Tuning of Surface Quenching for Luminescence Enhancement in Core–Shell Lanthanide-Doped Nanocrystals. *Nano Lett.* **16**, 7241–7247 (2016).

28. Wang, P. *et al.* 3.5 μm emission in Er3+ doped fluoroindate glasses under 635 nm laser excitation. *J. Lumin.* **237**, 118200 (2021).

29. Zhang, C., Zhang, C., Yun, C. & Lai, S. Intense broadband 3.1 μm emission in Er3+-doped fluoroaluminate-tellurite glass for mid-infrared laser application. *Ceram. Int.* **48**, 29977–29981 (2022).

30. Onishi, T. & Krimm, S. Origins of Characteristic Bands in the Infrared Spectra of Isotactic

Polystyrene and Isotactic Poly (Ring-d5 Styrene). *J. Appl. Phys.* **32**, (1961).

31. Mark, J. E. *Physical Properties of Polymers Handbook*. (Springer New York, 1984).
32. Quach, A. & Simha, R. Pressure-volume-temperature properties and transitions of amorphous polymers; polystyrene and poly (orthomethylstyrene). *J. Appl. Phys.* **42**, (1971).
33. Wisser, M. D. *et al.* Strain-induced modification of optical selection rules in lanthanide-based upconverting nanoparticles. *Nano Lett.* **15**, 1891–1897 (2015).
34. Bustamante, A., Calixto, M. E. & Rivas-Silva, J. F. Judd-Ofelt parameters and energy transfer rates of α-NaYF4:Eu3+– a theoretical and experimental study. *Ceram. Int.* **49**, 41098–41105 (2023).
35. Vedam, K., Meyers, J. & Mariner, G. Variation of refractive index of polystyrene with pressure to 7 kbar. *J. Appl. Phys.* **47**, 2443–2446 (1976).
36. Zhang, X. *et al.* Refractive index and polarizability of polystyrene under shock compression. *J. Mater. Sci.* **53**, 12628–12640 (2018).
37. Runowski, M. *et al.* Lifetime nanomanometry - high-pressure luminescence of up-converting lanthanide nanocrystals - SrF2:Yb3+,Er3. *Nanoscale* **9**, 16030–16037 (2017).

Supplementary Videos

Available at https://doi.org/10.25740/ff923hb3417

Supplementary Video 1 - Microgauge cross sections: Internal cross sections of a microgauge taken in a focused ion beam scanning electron microscope after platinum deposition (outer white layer). The polystyrene appears black and the UCNPs embedded inside appear as white dots.

Supplementary Video 2 - Microgauge transport under pharyngeal pumping: A video (66 fps) of microgauge transport under the influence of pharyngeal pumping in the terminal bulb. Dual illumination from a lamp (above) and a 980 nm laser (below) was used to make both the pharynx and the microgauges visible. Microgauge UCL is false colored in cyan. Scale bar is 50 μm.

Supplementary Video 3 - Microgauge ingestion in a freely crawling worm: A worm crawling on a lawn of microgauges and *E. coli* ingesting material and transporting it through the pharyngeal lumen to the intestines. The pharynx is facing in the direction of movement. Video has been slowed down 2.5 times. Scale bar, 250 μm.

Supplementary Video 4 - Microgauge defecation in a freely crawling worm: A worm crawling on a lawn of microgauges and *E. coli* undergoing a single defecation motor program cycle to expel material. Material can be seen moving anteriorly under the influence of a posterior body contraction then posteriorly under the influence of an anterior body contraction before the sphincter muscle rapidly and briefly contracts to expel material. The pharynx is facing left for the duration of the video. Video has been slowed down 2.5 times. Scale bar is 250 μm.

Supplementary Video 5 - Microgauge defecation in a channel immobilized worm: A UCL video (10x, 50 fps, 2s duration) of microgauge transport in the posterior intestinal lumen under the action of the defecation motor program. The approximate onset of posterior body contraction (pBoc), anterior body contraction (aBoc) and expulsion (Exp) events are labelled in the appropriate frames. Scale bar is 50 μm.

Supplementary Video 6 - Full segmentation example: A red UCL video (50x, 50 fps, 60s duration) of microgauges during pharyngeal pumping. The outline indicates the bounds of the pixel segment used to calculate the optical time series. This is the same dataset used to calculate the event-triggered averages shown in Fig. 4c.iv. We note that, out of the 30 pumps identified from the corresponding EPG, only 12 had noise levels low enough to meet our quality control standards (see Supplementary Fig. 16 for those that did not). Scale bar is 20 μm.
